# An Endosperm-Associated Cuticle Is Required for Arabidopsis Seed Viability, Dormancy and Early Control of Germination

**DOI:** 10.1371/journal.pgen.1005708

**Published:** 2015-12-17

**Authors:** Julien De Giorgi, Urszula Piskurewicz, Sylvain Loubery, Anne Utz-Pugin, Christophe Bailly, Laurent Mène-Saffrané, Luis Lopez-Molina

**Affiliations:** 1 Department of Plant Biology and Institute for Genetics and Genomics in Geneva (iGE3), University of Geneva, Geneva, Switzerland; 2 Developmental Biology Laboratory, Université Pierre et Marie Curie, Paris, France; 3 Department of Plant Biology, University of Fribourg, Fribourg, Switzerland; Harvard University, UNITED STATES

## Abstract

Cuticular layers and seeds are prominent plant adaptations to terrestrial life that appeared early and late during plant evolution, respectively. The cuticle is a waterproof film covering plant aerial organs preventing excessive water loss and protecting against biotic and abiotic stresses. Cutin, consisting of crosslinked fatty acid monomers, is the most abundant and studied cuticular component. Seeds are dry, metabolically inert structures promoting plant dispersal by keeping the plant embryo in an arrested protected state. In *Arabidopsis thaliana* seeds, the embryo is surrounded by a single cell endosperm layer itself surrounded by a seed coat layer, the testa. Whole genome analyses lead us to identify cutin biosynthesis genes as regulatory targets of the phytohormones gibberellins (GA) and abscisic acid (ABA) signaling pathways that control seed germination. Cutin-containing layers are present in seed coats of numerous species, including Arabidopsis, where they regulate permeability to outer compounds. However, the role of cutin in mature seed physiology and germination remains poorly understood. Here we identify in mature seeds a thick cuticular film covering the entire outer surface of the endosperm. This seed cuticle is defective in cutin-deficient *bodyguard1* seeds, which is associated with alterations in endospermic permeability. Furthermore, mutants affected in cutin biosynthesis display low seed dormancy and viability levels, which correlates with higher levels of seed lipid oxidative stress. Upon seed imbibition cutin biosynthesis genes are essential to prevent endosperm cellular expansion and testa rupture in response to low GA synthesis. Taken together, our findings suggest that in the course of land plant evolution cuticular structures were co-opted to achieve key physiological seed properties.

## Author Summary

Seeds are remarkable plant structures that appeared late during land plant evolution. Indeed, within seeds plant embryos lie in a metabolic inert and highly resistant state. Seeds allow plants to disperse and find a favorable living environment. Remarkably as well, the “near-dead” embryo is able to germinate and turn into a fragile young seedling. The fragility of this transition is betrayed by the existence of control mechanisms that block germination in response to harmful environmental conditions. Seeds therefore transform plants into time and space travellers and largely explain land plant colonization by flowering plants.

The key to this success lies in the seed’s physiological feats, a major yet unresolved question in plant biology. We show that mature seeds of the model plant Arabidopsis contain an earlier land plant evolutionary innovation: the cuticle, a waxy film covering the aerial parts of the plant preventing excessive transpiration. The seed cuticle, which contains cutin, a major lipid polymer component of the leaf cuticle, encloses all the living tissues within the seed. Seeds with cutin defects are highly oxidized and have low seed viability and dormancy. They are also unable to control their germination. Thus, land plants co-opted an ancient innovation to achieve the remarkable physiology of seeds.

## Introduction

Plant ancestors were aquatic organisms and their colonization of terrestrial habitats is a major chapter in the history of plant evolution. The cuticle and seeds are innovations that allowed plants to cope with lack of immediate water availability and a new gaseous environment.

The cuticle is a hydrophobic film covering aerial plant structures that appeared prior to seeds during land plant evolution. It limits transpiration and gas exchanges with the environment, while protecting the plant against pathogens and insects [1–3]. The cuticle consists of soluble and polymerized lipids. Cutin is a major cuticle component consisting of C_16_ and C_18_ oxygenated fatty acid monomers crosslinked with one another thus forming a polymeric hydrophobic network [4]. The first steps of cutin biosynthesis involve transfer of acyl-CoA by long-chain acyl-CoA synthetase (LACS) to fatty acid monomers [5, 6]. Thereafter, cutin monomer precursors are enzymatically modified by a family cytochrome P450 (CYP) family of fatty acil ω-hydroxylases proteins and of glycerol-3-phosphate O-acyltransferases (GPAT) that transfer hydroxyl groups and glycerol groups, respectively [4, 7, 8]. *DEFECTIVE IN CUTICULAR RIDGES* (*DCR*) encodes an acyltransferase incorporating cutin monomers into cutin polymers [9]. *BODYGUARD1* (*BDG1*) encodes a member of the alpha/beta hydrolase family that is important for final cutin organization [10]. The function of BDG1 remains unclear although it may be involved in the polymerization of cutin monomers.

Seeds are capsules enclosing plant embryos in a dry, metabolic inert and highly osmotolerant state. Seeds enhance plant exposure to new ecological niches by allowing plant dissemination. In the mature dry seeds of *Arabidopsis thaliana*, the embryo is surrounded by a single-celled layer of endosperm and an outer layer of dead tissue of maternal origin, the testa.

Imbibition with water is necessary but not sufficient for Arabidopsis seed germination to occur. Indeed, germination is tightly controlled by seed age or by environmental conditions upon imbibition [11]. Importantly, germination arrest protects the embryo by maintaining the seed’s osmotolerant state. Thus, germination arrest can be viewed as a protective mechanism [12, 13].

Seed dormancy, a trait of newly produced seeds, is the repression of germination under conditions normally favorable for seed germination. Dormancy prevents germination out of season and competition among siblings since it increases the chances of seed dispersion. Seeds lose dormancy after a period of dry after-ripening and become non dormant, i.e. they have the capacity to germinate upon imbibition. How dry after-ripening eventually breaks seed dormancy remains unknown. However, accumulation of oxidation events is known to tightly correlate with loss of dormancy. Furthermore, treatments that enhance seed oxidation accelerate loss of dormancy [14–16]. Seeds are very resistant structures but they nevertheless eventually lose viability, i.e. they fail to germinate upon imbibition [17]. Unsurprisingly, seed oxidation is also positively linked with loss of seed viability [18–20]. In some cases it was shown that seed’s internal structures are hypoxic environments, consistent with the notion that seeds limit oxygen diffusion to extend their viability [21, 22].

After-ripened, i.e. non-dormant seeds retain the capacity to control their germination. Indeed, upon imbibition, unfavorable light, osmotic potential or temperature conditions elicit seed germination arrest responses.

Seeds control their germination through the phytohormones gibberellic acid (GA) and abscisic acid (ABA) [11, 23, 24]. Unfavorable environmental conditions repress GA synthesis, which triggers overaccumulation of GA response DELLA factors such as RGL2. In turn RGL2 promotes endogenous ABA accumulation. ABA stimulates the accumulation and activity of ABA response factors that repress germination [25, 26]. This notably includes the basic leucine zipper (bZIP) transcription factor (TF) ABI5 [27–29]. Accordingly, *rgl2* and *abi5* mutant seeds are able to germinate in absence of GA biosynthesis [26]. Importantly, ABA-response factors, such as ABI5, maintain seed osmotolerance by stimulating *de novo* the expression of late seed maturation genes, including *LATE EMBRYOGENESIS ABUNDANT* (*LEA*) genes [13, 30].

Numerous studies have shown that seed coats of various plant species have fat-containing layers that include cutin or cutin-like depositions [31] (and references therein). Examples include layers present in seed coats of leek, onion, rapeseed and soybeen [31–34]. In developing Arabidopsis seeds, Molina et al provided biochemical and genetic evidence suggesting the existence of a cutin-like layer associated with the inner seed coat [32, 35]. These studies have either suggested or directly demonstrated the role of cutin-containing layers in regulating the seed’s permeability [31, 32, 34]. Furthermore, Arabidopsis mutant seeds specifically deficient in cutin biosynthesis genes can be affected in seed mucilage release, which regulates seed hydration properties, and germination ability under water-limiting conditions [9].

Suberin is another important plant wax also derived from fatty acids mostly present in outer bark and roots. Suberin early biosynthesis involves enzymes also involved in cutin biosynthesis [4]. Seeds of various plant species also contain suberin depositions including tomato, pepper, rapeseed and Arabidopsis [31, 32, 35–37]. In Arabidopsis, suberin biosynthesis mutant seeds have permeability alterations [36].

Taken together, these studies strongly indicate that lipid polymer layers must play fundamental roles in mature seed physiology and control of seed germination.

We sought to identify genes important for maintaining the arrested seed state upon imbibition. Whole-genomic approaches to identify RGL2-dependent gene expression and ABI5 gene occupancy lead us to identify cutin biosynthesis gene expression as being repressed in arrested seeds. This led us to further investigate the role of cutin in Arabidopsis seeds. Histological analysis identified an electron-dense cuticle layer similar to that found in leaf epidermis but about 10 times thicker, covering the outer face of endosperm cells in mature seeds. This endospermic cuticle displays morphological abnormalities in *bdg1* mutant seeds, which is associated with alterations in *bdg1* endosperm permeability. This strongly suggests that extra-embryonic cutin or cutin-like structures protect the plant’s living tissues within the mature seed. We show that cutin biosynthesis genes play an essential role to confer normal dry seed vitality and dormancy. We show that this correlates with higher seed oxidation events in cutin biosynthesis mutant seeds. We show that cutin biosynthesis gene expression is repressed upon imbibition when seeds are arrested. This suggests that de novo cutin biosynthesis gene expression is not necessary to repress germination. However, cutin biosynthesis mutants are unable to repress testa rupture when GA levels are low. This therefore strongly suggests that cutin biosynthesis gene expression played a central role during embryogenesis for the proper development of the mature seed. In turn, cutin depositions within the mature seed are needed to prevent testa rupture over time in imbibed seeds under low GA conditions.

## Results

### GA- and ABA-dependent cutin biosynthesis gene expression

To gain more insight on how GA and ABA signaling pathways control seed germination, we sought to identify genes whose expression is regulated by both RGL2 and ABI5.

**Fig 1:**
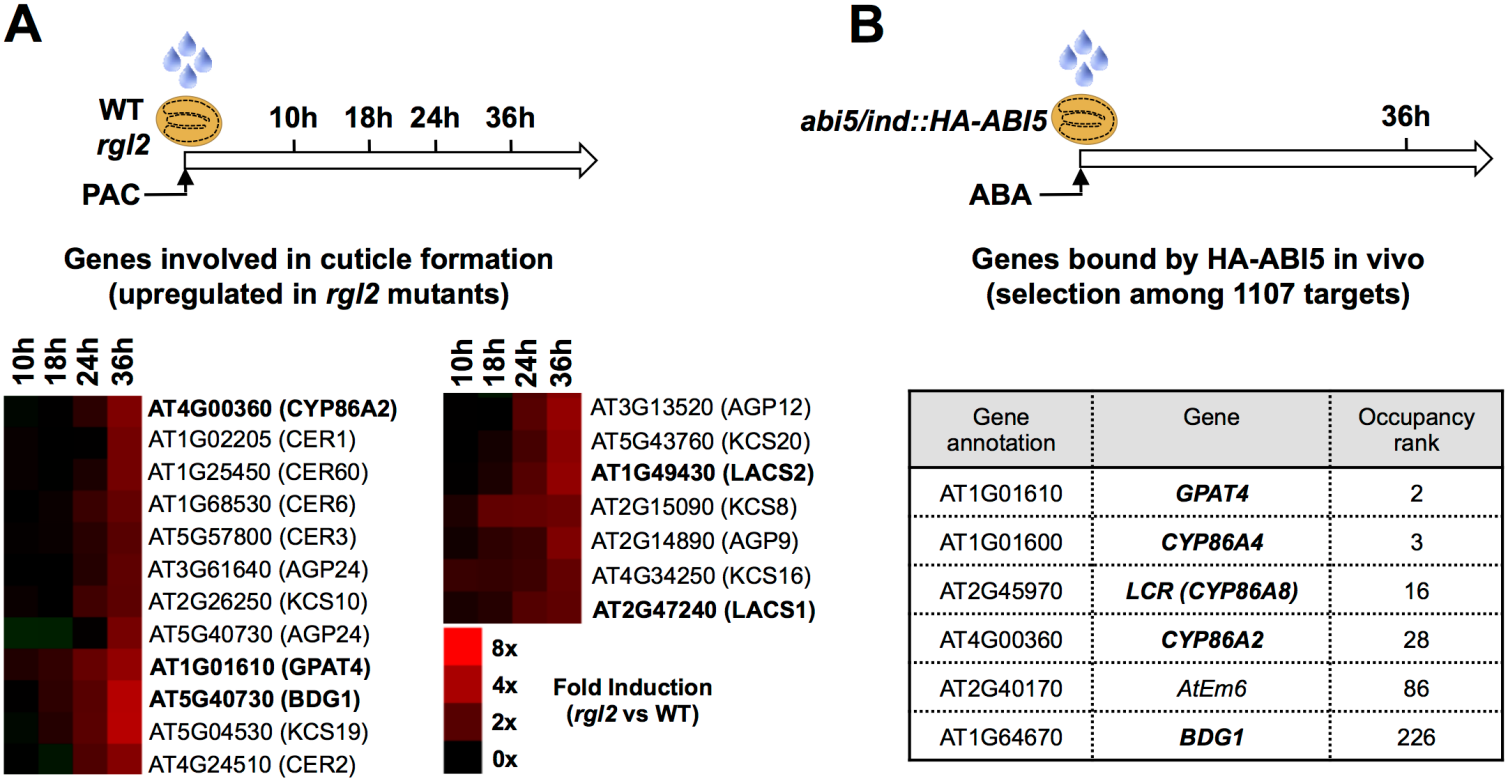
Cutin biosynthesis genes are potential targets of RGL2 and ABI5. (A) For whole genome expression studies (microarray), WT (Col) and *rgl2* seeds were harvested 10, 18, 24 and 36 hours after imbibition under low GA conditions (PAC). Dynamics of cuticle formation gene expression between *rgl2* and WT seeds is represented with a color code. The scale bar relates color with absolute fold changes. Genes in bold are involved in cutin synthesis. (B) For the ChIP-Seq experiment, seeds of *abi5-4/ ind*:: *HA-ABI5* were imbibed in the presence of estradiol and ABA and harvested 36 hours after imbibition [39]. The table shows cutin biosynthesis genes with high HA-ABI5 gene occupancy.

Whole-genome mRNA accumulation was investigated in WT and *rgl2* mutant seeds treated with paclobutrazol (PAC), an inhibitor of GA biosynthesis (Fig 1A). PAC treatment blocks WT seed germination but not *rgl2* mutant seed germination [25]. To identify early RGL2-dependent gene expression, RNA was isolated from seed material harvested at different time points early upon seed imbibition before *rgl2* mutant seed germination (Fig 1A). RNA was used to compare the transcriptomes of WT and *rgl2* seeds by Affymetrix microarray hybridization (Materials and Methods). 36 hours after imbibition, the analysis revealed 382 and 457 transcripts down- and up-regulated (more than twofold), respectively, in *rgl2* (S1 Table and S1 File). Mapman software analysis revealed that among the transcripts up-regulated in *rgl2*, as much as 45 were involved in primary cell wall modifications and 19 were involved in cuticle formation (Fig 1A, S1 Fig) [38].

Concerning genes regulated by ABI5, we sought to identify genes bound by ABI5 in vivo as potential genes directly regulated by ABI5. We used an *abi5-4* mutant line transformed with an estradiol-inducible promoter driving the expression of hemagglutinin (HA)-tagged ABI5 (*abi5-4*/*ind*::*HA-ABI5*) (Fig 1B). We previously used this line for chromatin immunoprecipitation coupled with massively parallel DNA sequencing (ChIP-Seq) to report 9 genes bound by ABI5 in vivo [39]. Here we report 1107 genomic sequences associated with HA-ABI5 more than 20 fold above the background signal (Materials and Methods) [39]. They are ranked in a descending order according to the levels of HA-ABI5 occupancy (Fig 1B and S2 Table).

Strikingly, we found that within the first 30 genomic sequences potentially bound by ABI5 as much as 4 were associated with genes involved in cutin biosynthesis (Fig 1B). *GPAT4* (ranked #2 in the list) encodes a sn-glycerol-3-phosphate 2-O-acyltransferase/phosphatase [7, 40]. *CYP86A4* (rank #3), *CYP86A8* (*LACERATA -LCR-* rank #16) and *CYP86A2* (rank #28) encode fatty acyl ω-hydroxylases [32, 41–43]. ABI5 occupancy levels of these genes were higher than well-established ABI5 target genes, such as *AtEm6* (rank #87) [13, 30]. Furthermore, *BDG1*, encoding a putative alpha/beta-hydrolase also involved in cutin biosynthesis in an unknown manner, was ranked at position #227 [10].

*GPAT4*, *CYP86A2* and *BDG1* were also listed among genes whose expression is upregulated in *rgl2* mutants (Fig 1A). This was also the case of *LACS2* and *LACS1*, both encoding a long chain acyl-CoA synthetase (LACS) involved in cutin biosynthesis [5, 6, 44].

### A thick cuticular layer covers the endosperm on its outer side

The whole-genome data analysis presented above suggested that low GA and high ABA levels repress de novo cutin biosynthesis gene expression in imbibed arrested seeds. The role of cutin within cuticular structures already present in mature seeds plays is poorly understood.

To address this question, we first sought to assess the presence of cuticular structures in Arabidopsis mature seeds.

**Fig 2:**
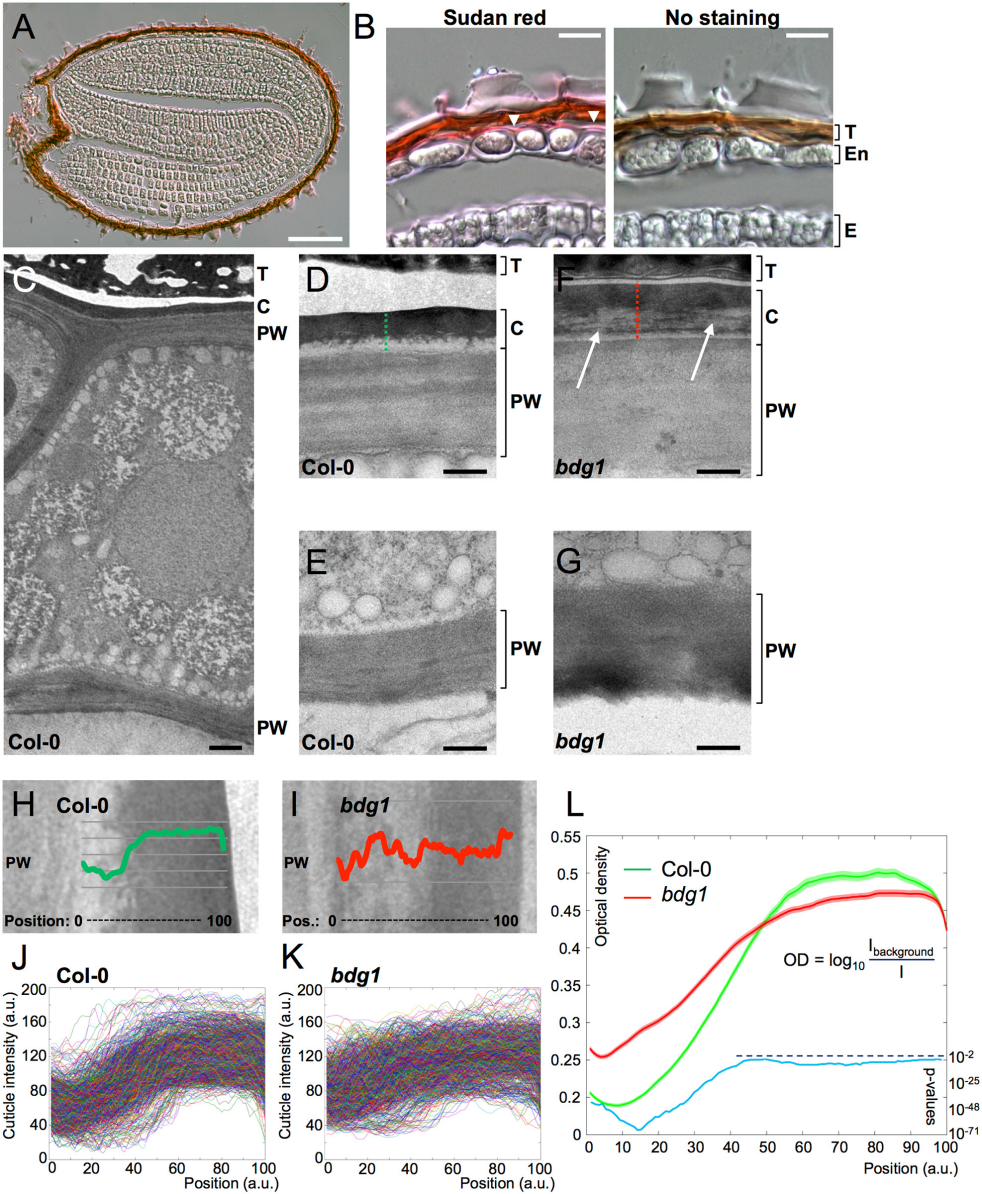
A cuticle is present on the outer side of endosperm cells. (A) Sudan Red staining of a Col-0 seed section. Bar: 100μm. (B) Close-up of a Col-0 seed section stained with Sudan Red. The pink line between the endosperm cell bodies and the testa (arrowheads) indicates the presence of a cuticle on the outer side of endosperm cells. This pink line is specific to the Sudan Red staining, as it is not seen in unstained seed sections (right panel). T: testa; En: endosperm; E: Embryo. Bars: 15μm. (C) Electron micrograph of a Col-0 endosperm cell. Bar: 1000nm. (D-G) Close-ups of the outer (D, F) and inner (E, G) sides of endosperm cells in Col-0 seeds (D, E) and *bdg* seeds (F, G). Note the presence of a cuticle exclusively on the outer side of endosperm cells. The arrows in (F) indicate defects in the cuticle of a *bdg1* cell. Abbreviations are: T: testa; C: cuticle; PW: primary wall. Bars: 300nm. (H-I) Profiles of the cuticle optical intensity along the dotted lines in (D) and (F), respectively. (J-K) Profiles of cuticle optical intensity in all the regions analysed in Col-0 cells (J; 969 lines) and *bdg1* cells (K; 1002 lines). See the Materials and Methodssections for details on the measurement procedure. (L) Average cuticle optical density in Col-0 cells (green line) and *bdg1* cells (red line). Standard errors of the mean are represented as shaded areas around each line. The blue line represents (on a semi-log scale) the p-values given by Kolmogorov-Smirnov tests performed at each position along the cuticle between the Col-0 and the *bdg1* cuticle densities; the dark blue dotted line represents the 10^−2^ threshold of significance for these p-values.

In exploratory experiments, Sudan Red dye, which specifically stains in pink or red wax and lipids, was used in seed histological sections (Fig 2A). As a guiding reference, we stained WT leaf sections to visualize the leaf cuticle. Sudan Red stained the outer layer of the adult leaf epidermis, consistent with its ability to recognize cuticle components (S2 Fig). This indicated that Sudan Red could be used to identify cutin or cutin-like depositions in seeds. In WT seed sections, we could observe a smooth extracellular pink stained layer on the outer side of endosperm cells (Fig 2B). This layer was not visible on the inner side of the endosperm nor on the surface of the embryo. These experiments therefore suggested that a cutin-containing cuticle surrounds the entire outer side of the endosperm, i.e. the entire living seed tissues.

To strengthen the notion that this layer is a cutin-containing structure, we performed electron microscopy. Consistent with the Sudan Red staining, a smooth, thick and electron-dense cuticle covering the outer primary wall of endosperm cells was observed (Fig 2C and 2D **)**. This cuticular structure was most similar to that reported in leaves but, strikingly, was nearly 10 times thicker than the cuticle present on the leaf surface (315+/-9.8nm in seeds, n = 39 micrographs; 33+/-5.2nm in leaves, n = 9 micrographs; averages +/- standard errors of the mean, see Materials and Methodsfor details). This thickness was about 34+/-1.5% relative to that of the primary cell wall, compared to 8.6+/-1.1% in leaves (averages +/- standard errors of the mean). Furthermore, the endospermic cuticle surrounded the whole endosperm (micropylar, peripheral and chalazal) and could be found only in its outer face, i.e. on the side facing the testa but not on the side facing the embryo (Fig 2C–2E). We also examined *bdg1* mutant seeds given their known morphological cuticular abnormalities in leaves [10]. As expected, in *bdg1* seeds, the endospermic cuticle could only be observed covering the outer primary wall of endosperm cells (Fig 2F and 2G). However, its internal appearance displayed cavernous and filamentous structures of lower electronic density, reminiscent of what can be observed in the *bdg1* leaf cuticle (Fig 2F and S3 Fig) [10]. Quantification of the optical density distribution throughout the cuticle confirmed the occurrence of an abnormal cuticle in *bdg1* mutant seeds (Fig 2H–2L). In particular, the disorganization of the *bdg1* cuticle translated into a higher average optical density in the proximity of the primary cell wall (Fig 2H–2L).

We did not observe a clear, electron dense cuticular layer surrounding the mature embryo. S4 Fig shows a representative example of what could be the clearest evidence indicating the occurrence of an embryonic cuticle (S4 Fig). For size comparison, an inset shows the cuticle associated with the endosperm within the same electron microscopy section. Even in this case, it appears that a putative embryonic cuticle would not be comparable in thickness to that observed in the outer PW of endosperm cells.

Taken together, these observations strongly suggest that a cuticular layer containing cutin or cutin-like depositions covers the endosperm on the side facing the testa.

These findings prompted us to further explore the role of cutin in germination control responses and seed physiology using a genetic approach.

### Cutin biosynthesis mutants fail to block endosperm cell expansion under low GA conditions

**Fig 3:**
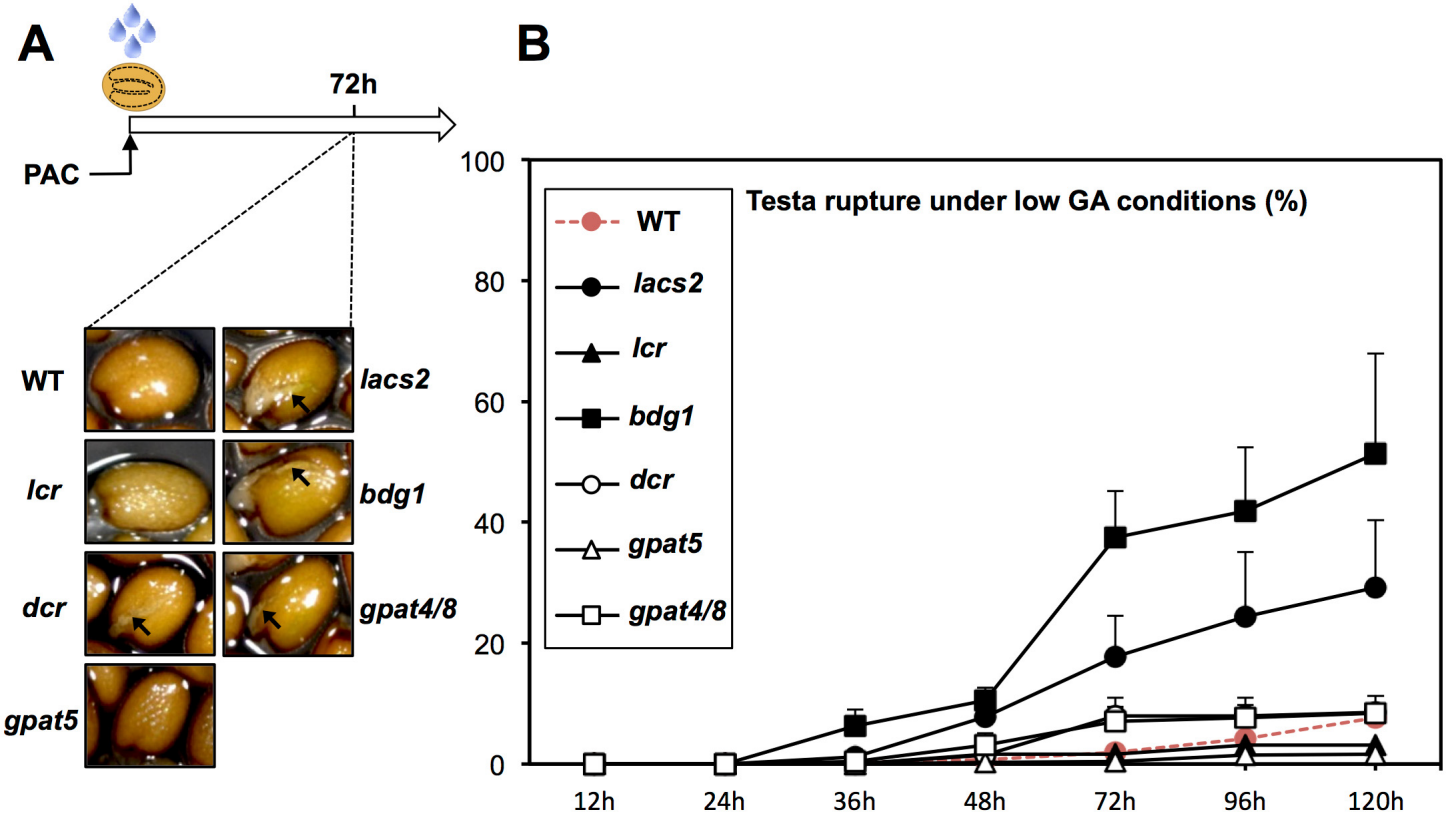
Imbibed seeds of cutin biosynthesis mutants can rupture testa under low GA conditions. (A) Images showing WT (Col), *lacs2*, *lcr*, *bdg1*, *dcr*, *gpat4/8*, *gpat5* seeds 72h after imbibition under low GA conditions. Black arrows indicate testa rupture. (B) Chart represents percentages of testa rupture over time (in hours) under low GA conditions (4 replicates (*n* = 150–200)).

We monitored the germination of cutin biosynthesis mutant seeds under normal conditions and in presence of PAC (low GA conditions). *Arabidopsis* seed germination first involves testa rupture (TR), about 24 hours after seed imbibition. TR is soon followed by endosperm rupture (ER), which occurs concomitantly with radicle protrusion out of the seed. The ER event is often referred as germination *sensu stricto* [45]. Mature cutin biosynthesis mutant seeds had normal appearance. Under normal germination conditions, none of the mutant seeds displayed significant differences in the percentage of testa and endosperm rupture events when compared to WT seeds (S5A Fig). In contrast, *lacs2*, *bdg1* and, to a lesser extent, *gpat4/gpat8* and *dcr* mutants were able to rupture testa under low GA conditions that fully prevent testa and endosperm rupture of WT seeds (Fig 3). However, TR was normally prevented in *lcr* mutants. Endosperm rupture could also be observed in cutin biosynthesis mutants but to a lower extent (S5B Fig). Suberin is another important fatty-acid plant polymer and *gpat5* mutants are deficient in suberin synthesis [4, 36]. *gpat5* mutant seeds displayed normal germination responses under normal and low GA conditions (S5A Fig and Fig 3). Taken together, these observations strongly suggest that cutin can be involved in maintaining repression of TR under low GA conditions.

**Fig 4:**
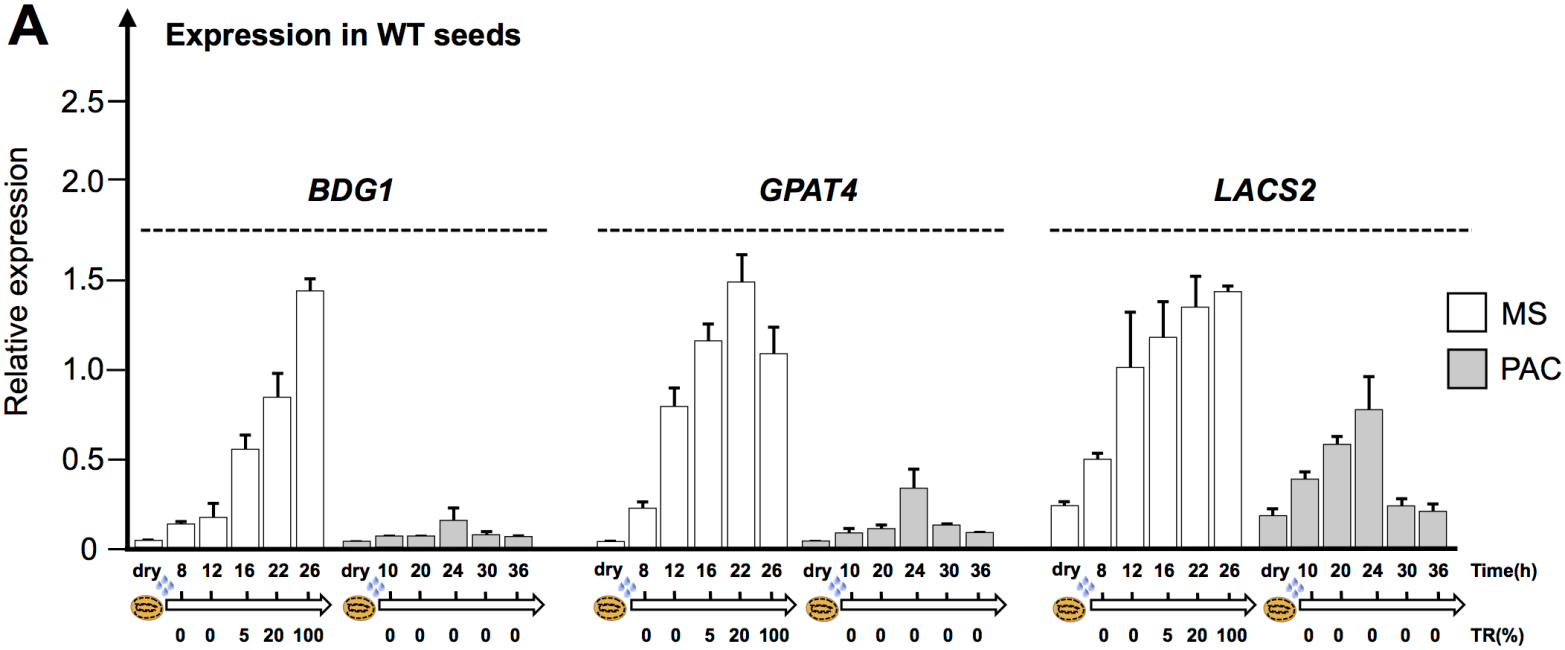
Expression of cutin biosynthesis genes is low when GA synthesis is inhibited. (A) Histograms show the relative *BDG1*, *GPAT4* and *LACS2* mRNA accumulation in WT (Col) dry seeds as well as 8h, 12h, 16h, 22h and 26 h (MS) and 10h, 20h, 24h, 30h and 36 h (PAC) and after imbibition under normal and low GA conditions (PAC).

To better understand this phenotype, we sought to further assess the temporal expression of *BDG1*, *GPAT4* and *LACS2* upon WT seed imbibition under normal and low GA conditions. *BDG1*, *GPAT4* and *LACS2* expression was low in dry seeds and increased 12h after seed imbibition, i.e. prior to TR, and thereafter until 26h after imbibition when 100% of seeds had ruptured their testa (Fig 4). In contrast, when seeds were imbibed under low GA conditions, *BDG1*, *GPAT4* and *LACS2* expression remained lower over time reaching levels similar to that in dry seeds. These data therefore indicate that de novo cutin biosynthesis is not necessary to block TR under low GA conditions.

It has been proposed that TR is initially driven by specific expansion of micropylar cells, which is associated with cellular water uptake [46, 47]. We sought to explore whether the occurrence of TR in *lacs2* and *bdg1* mutants under low GA conditions is associated with abnormal micropylar endosperm cellular expansion.

**Fig 5:**
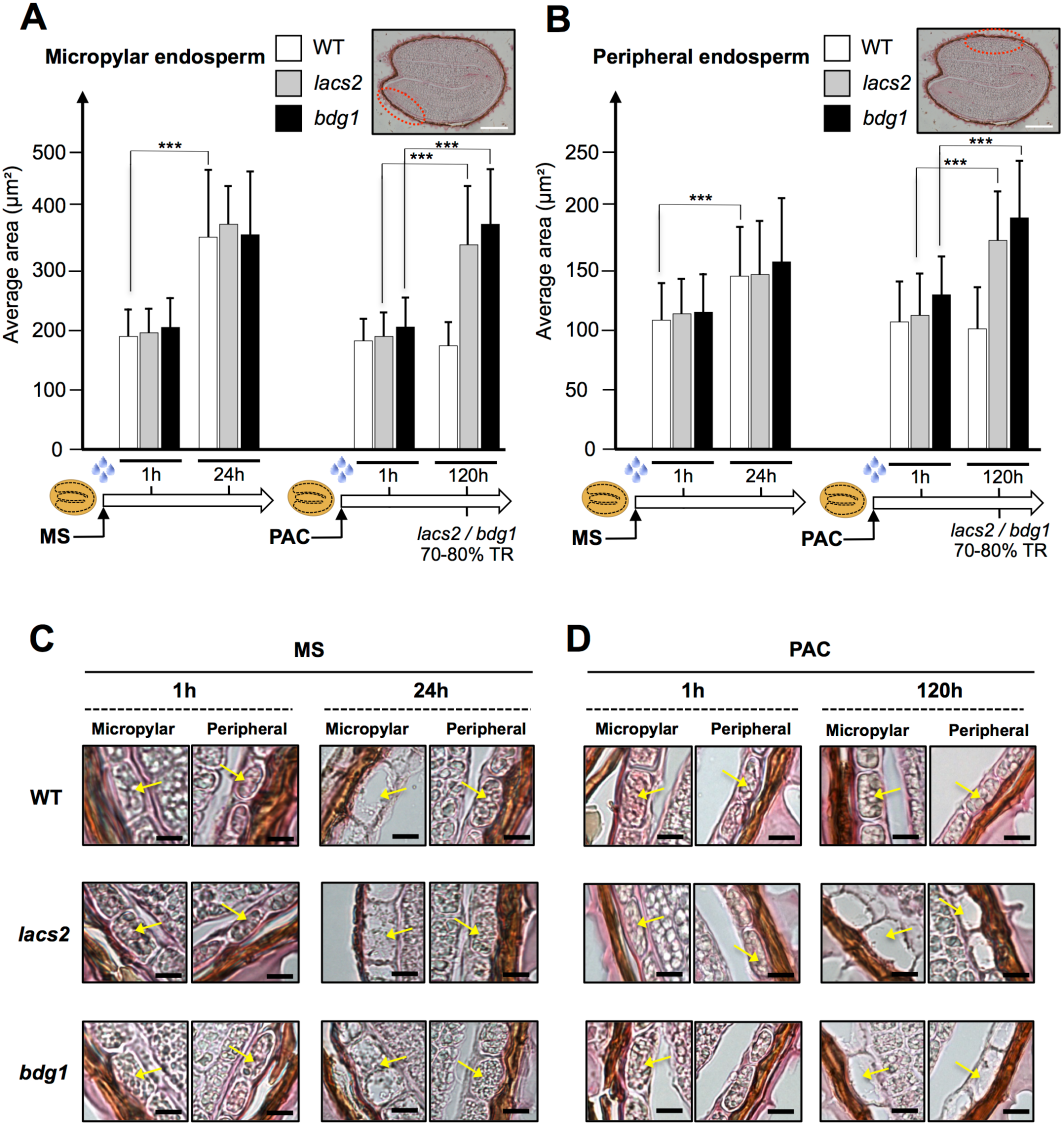
Testa rupture correlates with the expansion of the micropylar endosperm cells. *bdg1* and *lacs2* mutants fail to arrest micropylar endosperm cell expansion when GA synthesis is inhibited. (A) Histograms show the average area of micropylar endosperm cells in WT (Col), *lacs2*, *bdg1* seeds at 1 and 24 hours after imbibition under normal conditions (MS) and 1 and 120 hours after imbibition under low GA conditions (PAC). (*** = P-values < 0.001, n equal 80 to 220 cells). Bar: 200μm. (B) Histograms show the average area of peripheral endosperm cells in WT (Col), *lacs2* and *bdg1* seeds at 1and 24 hours after imbibition under normal conditions (MS) and 1and 120 hours after imbibition under low GA conditions (PAC). (*** = P-values < 0.001, n equal 70 to 150 cells). The same seeds used in (A) were used for peripheral endosperm measurements. Bar: 200μm. (C) Representative pictures of micropylar and peripheral endosperm cells of WT (Col), *lacs2* and *bdg1* taken at 1 and 24 hours after imbibition under normal conditions (MS). Yellow arrow indicates a cell of the endosperm. Bars: 10μm. (D) Same as (C) with pictures taken 1 and 120 hours after imbibition under low GA conditions (PAC).

We measured the area of micropylar and peripheral endosperm cells in WT, *lacs2* and *bdg1* seed histological sections (Materials and Methods). Under normal conditions, seeds were harvested 1h after seed imbibition and 24h thereafter, i.e. prior to visible testa rupture. Under these conditions, the area of micropylar endosperm cells expanded by about 85% (p<0.001) whereas that of peripheral endosperm cells by only 32% (p<0.001) (Fig 5A and 5B). One hour (1h) after seed imbibition, the interior of micropylar endosperm cells appeared densely stained (Fig 5C). In contrast, 24h after imbibition, micropylar endosperm cells became less densely stained and exhibited larger inner transparent spaces. These changes were less evident in peripheral endosperm cells (Fig 5C). Altogether, these observations support the notion that micropylar endosperm cellular expansion positively correlates with testa rupture.

As expected, seed imbibition for one hour under low GA conditions was not sufficient to reveal significant differences in endosperm cell size between WT and *bdg1* or *lacs2* seeds (Fig 5A and 5B). After 120h, WT seeds did not rupture their testa while retaining the same intracellular appearance and cell size observed 1 hour after seed imbibition (Fig 5D). This further reinforces the view that micropylar endosperm cell expansion and testa rupture are positively correlated.

In marked contrast, over the same 120 hour period, *lacs2* and *bdg1* micropylar cells underwent a marked increase in cell size of about 90% (p<0.001), which was associated with 70–80% TR (Fig 5A). Cell expansion was also visible with peripheral endosperm cells, which underwent a 40% (p<0.001) increase in cell size (Fig 5B). Cell expansion was associated with an increase in intracellular transparency, which was due to the presence of intracellular vacuoles (Fig 5D).

Altogether, these observations show that *lacs2* and *bdg1* mutant seeds, deficient in cutin biosynthesis, are unable to block expansion of endosperm cells under low GA conditions. This is particularly the case of micropylar endosperm cells.

To explore whether these phenotypes could correlate with changes in the permeability of the cuticle we compared the uptake of the toluidine blue dye in WT and *bdg1* seeds arrested by PAC [48].

**Fig 6:**
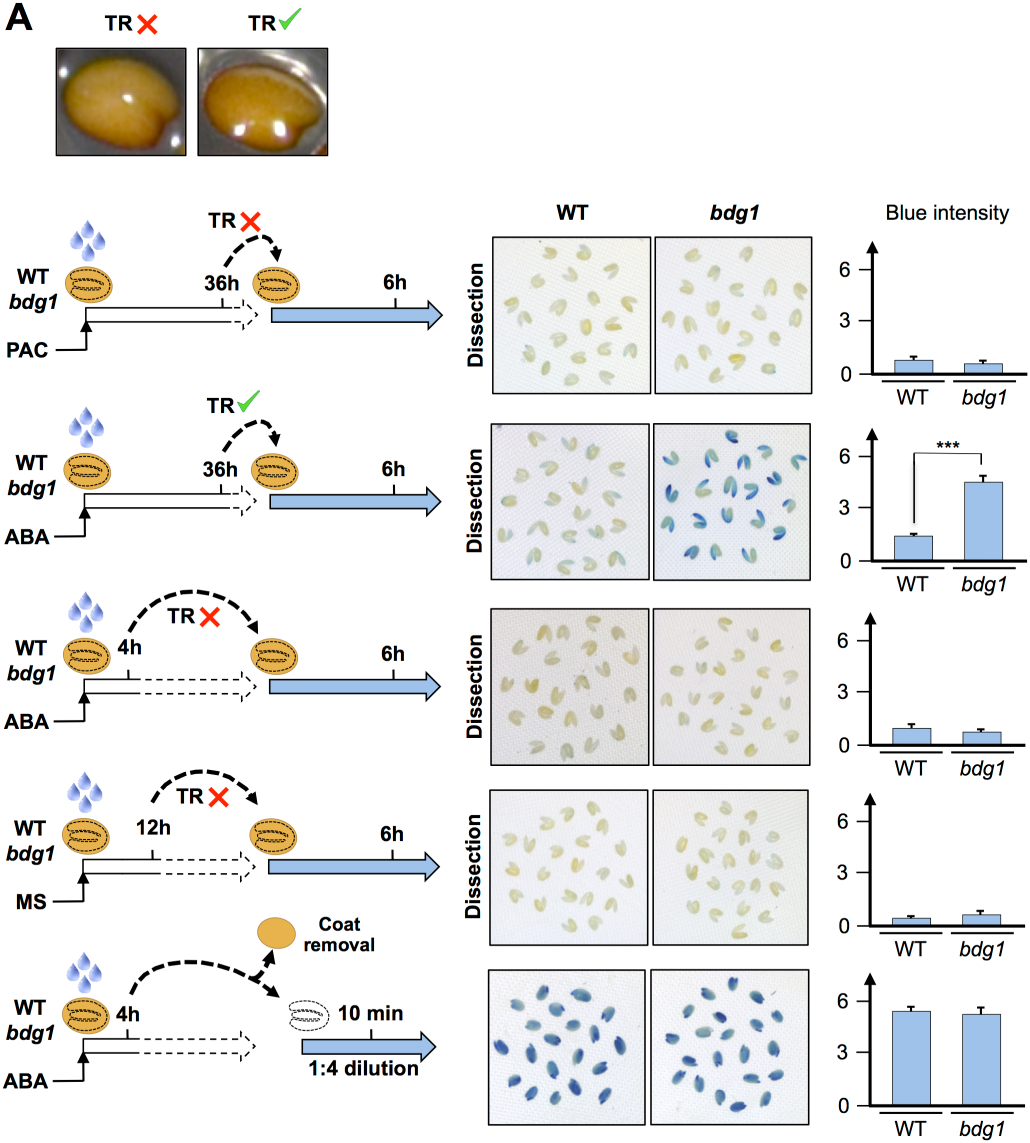
*bdg1* endosperm exhibits higher permeability to toluidine blue. (A) Comparison of toluidine blue staining in embryos dissected from WT and *bdg1* seeds. Whole seeds of WT and *bdg1* were incubated on MS, ABA and PAC for different times prior or after TR as indicated. Thereafter, seed material was transferred to a toluidine blue solution for 6 hours. The solution maintained PAC or ABA if these compounds were previously present in the germination medium. For illustration, a picture of a seed with or without TR is shown. Pictures show embryos dissected out of seed coats after 6h incubation in toluidine blue solution. Histograms show blue intensity quantification obtained from pictures (Materials and Methods). (*** = P-values < 0.001).

WT and *bdg1* seeds were cultured in presence of PAC for 36h, i.e. prior to TR in *bdg1* mutants thus allowing direct comparison of toluidine blue uptake in WT and *bdg1* seeds. Thereafter, WT and *bdg1* seeds were transferred to a toluidine blue solution also containing PAC and further incubated for 6h. These treatments did not lead to TR. We observed no significant toluidine blue coloration in both WT and *bdg1* dissected embryos suggesting that the toluidine blue dye did not penetrate the seed beyond the testa, which does not allow assessing endosperm’s permeability to the dye (Fig 6). Indeed, dissection of the testa and endosperm showed that all the blue dye was localized on the outer side of the testa while leaving the internal side, with the endosperm still attached, colorless (S6 Fig).

To circumvent this difficulty, we attempted to remove the testa while leaving the endosperm surrounding the embryo. However, testa dissection is very difficult to perform without generating small endosperm lesions that lead to rapid toluidine blue uptake by the embryo at the site of the injury.

We therefore considered the case of seeds incubated in presence of ABA for 36 hours. Indeed, in these conditions testa rupture takes place in both WT and *bdg1* seeds while endosperm rupture is prevented, thus exposing the endosperm to the outer environment [26, 49]. ABA-treated WT and *bdg1* seeds were selected after 36h of incubation so that they displayed similar extent of testa rupture (Fig 6). Remarkably, after 6h of incubation with toluidine blue solution, the *bdg1* embryos were markedly more stained than WT embryos (Fig 6). Furthermore, the staining was mostly localized to the radicle, which is the embryonic tissue most physically close to micropylar endosperm. In control experiments we did not observe significant WT or *bdg1* embryo coloration when seeds were incubated for a shorter time in presence of ABA, giving no time for testa rupture to take place (Fig 6). Similar results were obtained when seeds were imbibed under normal conditions for 14h, i.e. prior to TR (Fig 6).

We also compared toluidine blue uptake of dissected, coat-less WT and *bdg1* embryos. This necessitated diluting the toluidine blue solution to prevent very rapid and intense blue staining of both WT and *bdg1* embryos. Similar and rapid coloration was observed in both WT and *bdg1* embryos (Fig 6).

These experiments provide direct evidence that the endosperm of *bdg1* seeds is more permeable to the toluidine blue dye than the endosperm of WT seeds whereas WT and *bdg1* embryos are similarly permeable to the dye.

### Cutin biosynthesis mutants are more sensitive to oxidation

The leaf cuticle plays an important role in the plant’s relations with its gaseous environment. We therefore explored whether cutin biosynthesis regulates two important aspects of seed physiology: seed viability and dormancy. Indeed, both traits are known to be influenced by oxidation events.

**Fig 7:**
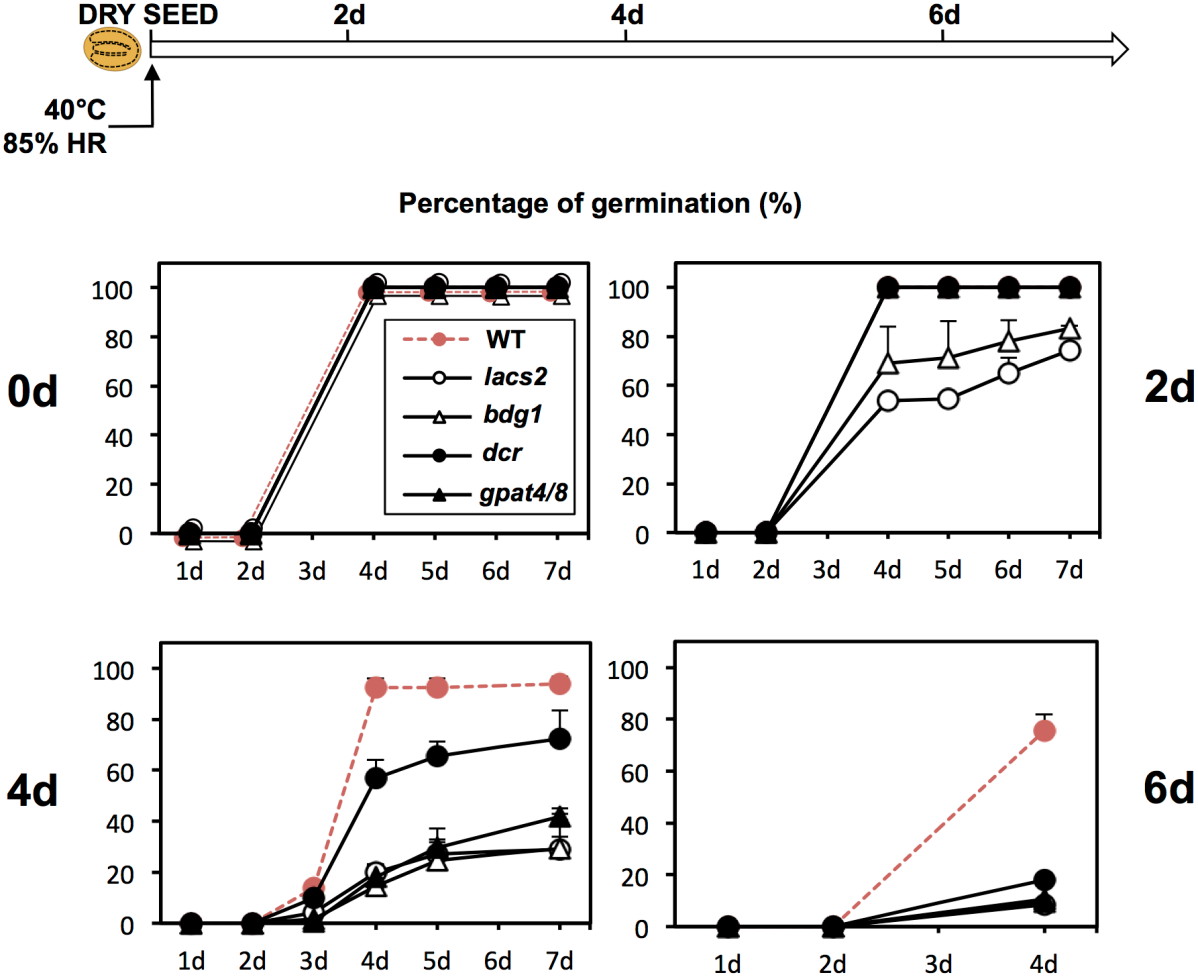
Dry seeds of cutin biosynthesis mutants lose viability faster than WT. An accelerated aging treatment is applied for 0, 2, 4 and 6 days on WT (Col), *lacs2*, *bdg1*, *dcr*, *gpat4/8*, *gpat5* dry seeds. Graphs represent a percentage of seeds germination over time (in days) (seeds were plated in triplicate (*n* = 100–150)). Before imbibition dry seeds underwent an accelerated aging treatment for 0, 2 and 4 days (indicated on the graphs). Seeds were germinating at 15°C in darkness.

Seed viability is the capacity of freshly produced seeds to survive in the dry state for extended periods of time while maintaining the potential to successfully germinate upon seed imbibition. Relative air humidity determines seed moisture content and influences seed viability by notably stimulating oxidation events that contribute to deteriorate seeds [14]. Seed viability can be assessed by subjecting seeds to an accelerated aging protocol where seeds are maintained at high temperature and high relative humidity [20]. This treatment was performed in WT seeds for 2, 4 and 6 days (Fig 6). After 4 days of treatment, all WT seeds retained the capacity to germinate but after 6 days, about 75% of WT seeds germinated implying that they were starting to die following the accelerated aging treatment (Fig 7).

In marked contrast, *bdg1* and *lacs2* mutant seed viability was already challenged after only 2 days of accelerated aging treatment since only about 80% of seeds germinated (Fig 7). After 4 days, *bdg1* and *lacs2* seeds further lost viability whereas *gpat4*/*gpat8* and *dcr* seeds started to lose viability. After 6 days of treatment, only about 15% of *lacs2*, *bdg1*, *gpat4/8* and *dcr* seeds germinated 4d after imbibition (Fig 7).

In conclusion, these data show that the cutin biosynthesis deficient mutants *bdg1*, *lacs*, *gpat4*/*gpat8* and *dcr* seeds exhibit low seed viability.

**Fig 8:**
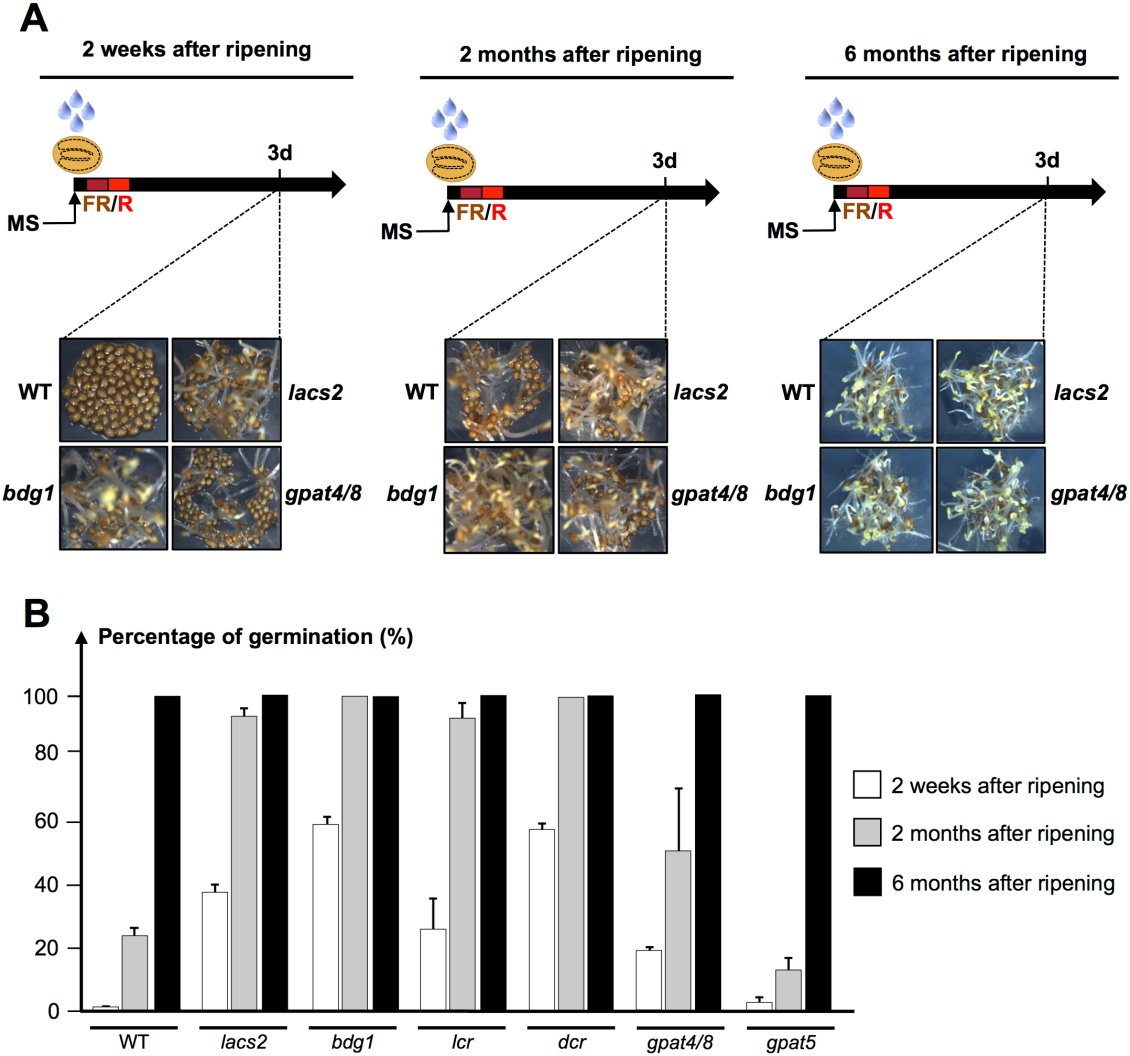
Cutin biosynthesis mutants exhibit low dormancy levels. (A) Germination is induced after a FR pulse (5min) is immediately followed by a R pulse (5min)(FR/R). WT (Col), *lacs2*, *bdg1*, *gpat4/8* seeds with different after ripening times were germinating in darkness and pictures were taken 3 days after FR/R treatment. (B) Histograms represent the percentage of germination of WT (Col), *lacs2*, *bdg1*, *lcr*, *dcr*, *gpat4/8*, *gpat5* seeds 3 days after imbibition under suboptimal germination conditions (seeds were plated in triplicate (*n* = 100–150)).

The ecotype Columbia used in this study is known to display low dormancy levels under standard germination conditions. Thus, freshly harvested Col-0 seeds normally acquire within a few days the capacity to germinate when sown under standard germination conditions. To better evaluate changes in dormancy levels, we used suboptimal germination conditions consisting of imbibing seeds in 1) darkness with a far red (FR) pulse immediately followed by a red (R) pulse and 2) darkness. After the FR/R pulses, WT seeds that underwent a period of after-ripening of 2 weeks were unable to germinate being therefore dormant (Fig 8A and 8B). However, after a period of 2 months of after-ripening, about 20% of WT seeds from the same seed batch were able to germinate (Fig 8A and 8B). Eventually, after 6 months of after-ripening, all WT seeds germinated and therefore lost their dormancy relative to the germination conditions used (Fig 8).

In contrast, *bdg1*, *lacs2*, *lcr* and *dcr* seeds after-ripened for 2 weeks and 2 months were clearly less dormant as they were able to germinate at a percentage superior to 20% and 80%, respectively (Fig 8A and 8B). Similarly, *gpat4/8* mutant seeds were less dormant but to a lesser extent than *bdg1*, *lacs2*, *lcr* and *dcr* seeds (Fig 8A and 8B). Interestingly, *gpat5* did not display any noticeable decrease in seed dormancy levels, consistent with previous reports (Fig 8B) [36]. This indicates that the observed accelerated loss in seed dormancy specifically arises from deficient cutin depositions in dry mature seeds.

Similarly, *bdg1*, *lacs2* and *gpat4/8* exhibited lower dormancy in darkness (S7A and S7B Fig).

Lower dormancy levels in cutin biosynthesis mutants suggested that deficiencies in cutin depositions mature seeds lead to increased oxidative events in seeds, thus explaining not only their lower viability but also their rapid loss of dormancy.

**Fig 9:**
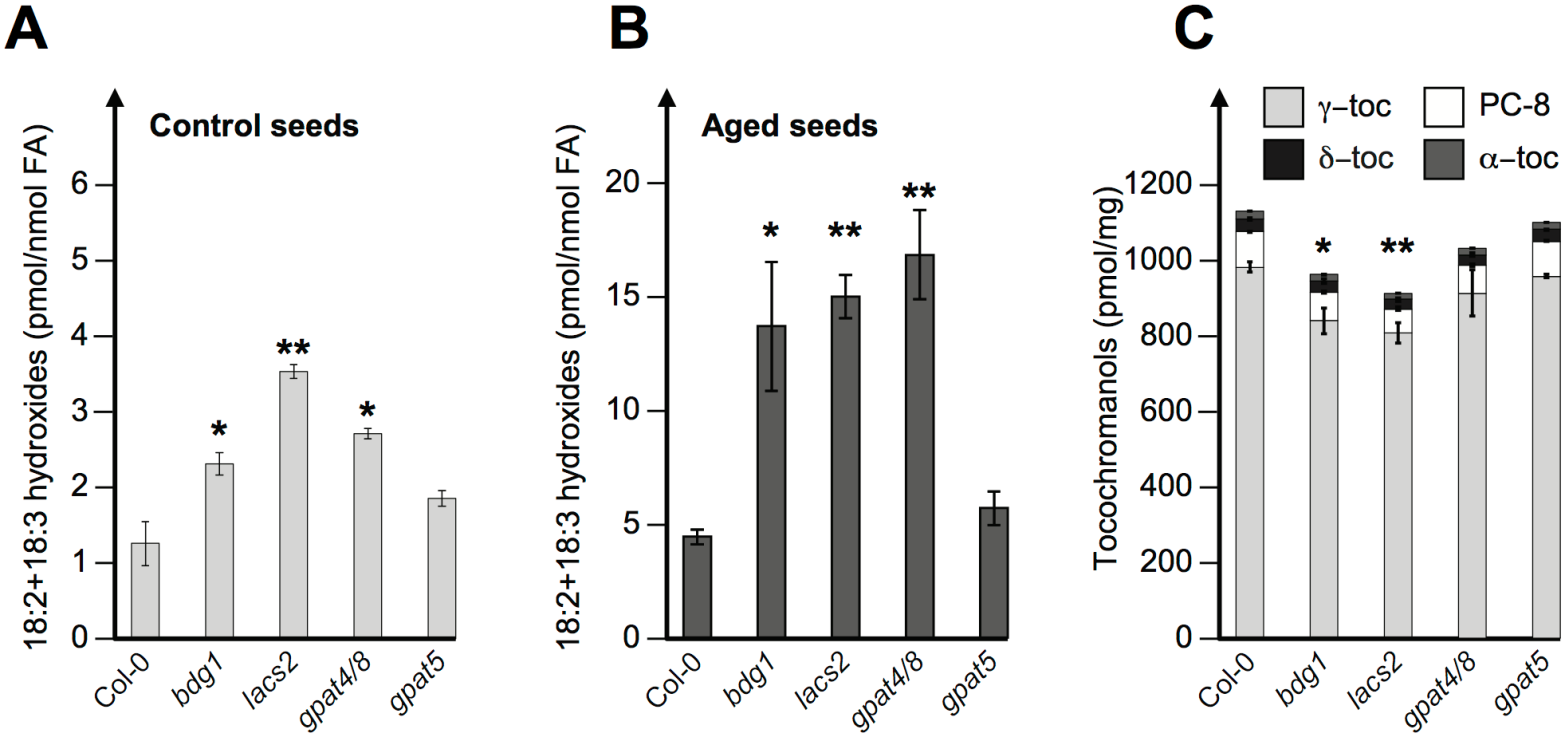
Polyunsaturated fatty acid hydroxides and tocochromanols in Arabidopsis cutin-deficient seeds. Seed lipid oxidation was measured by quantifying 9- and 13-hydroxy derivatives of linoleic and linolenic acids in untreated seeds (A) and seeds aged for 6 days (B). Data are expressed as pmol of hydroxy fatty acid per nmol of unmodified fatty acid (average±STDEV; n = 2). Tocochromanol levels in untreated seeds (average±STDEV; n = 2) (C). Asterisks represent significance levels using Student’s *t* test of each genotype relative to Col-0 WT controls. *: *P*<0.05; **: *P*<0.01).

To address this hypothesis we quantified the relative levels of stable markers of lipid oxidation, esterified fatty acid hydroxides (LOH). As expected *vte2/vte1* double mutant seeds, unable to synthesize the antioxidant tocopherol, accumulated markedly higher LOH levels, consistent with previous results (S8 Fig)[50]. Cutin biosynthesis mutants that did not receive an accelerated aging treatment had 2 to 3.5 fold higher LOH levels compared to WT seeds (Fig 9A). Consistent with our hypothesis, these fold differences persisted in seeds subjected to a 6 day accelerated aging procedure that stimulated LOH levels by about 4 fold (Fig 9B). In contrast, *gpat5* LOH levels remained comparable to those observed in WT seeds (Fig 9A and 9B). In addition, *bdg1* and *lacs2* seeds accumulated lower tocopherol levels than WT seeds, unlike *gpat5* seeds (Fig 9C). Lower tocopherol levels further suggest that *bdg1* and *lacs2* seeds have higher susceptibility to lipid oxidation.

Overall, these data confirm the notion that cutin biosynthesis genes protect seeds from oxidative damage, which correlates with the lower viability and dormancy of cutin biosynthesis mutant seeds.

### Embryonic cutin in mature seeds is necessary to prevent TR under low GA conditions

So far, this study focused on the seed physiological role of cutin depositions already present in mature seeds by studying cutin biosynthesis mutant seeds. However, this study was initiated after a whole genome analysis suggested that GA and ABA signaling pathways regulate cutin biosynthesis gene expression upon seed imbibition. We seek now to further clarify how GA and ABA signaling regulate upon seed imbibition 1) cutin biosynthesis gene expression and 2) TR.

Low GA levels lead to RGL2 protein overaccumulation and genetic analysis has shown that RGL2 is necessary to 1) repress TR and 2) promote accumulation of endogenous ABA levels [26]. It was initially proposed that ABA does not participate to repress TR since ABA applied exogenously does not significantly repress TR [26, 49, 51]. Consistent with this notion, germination of the ABA signaling *abi5* mutant is “explosive” under low GA conditions, i.e. ER takes place without prior visible TR [26]. However, ABI5 is not the only ABA-response factor in seeds and we observed that PAC-treated *aba1-6* mutants, unable to synthesize GA, display TR (S9 Fig). This shows that endogenous ABA synthesis is also necessary to repress TR under low GA conditions.

Thus, *lacs2*, *bdg1* and *gpat4/gpat8* testa rupture under low GA conditions could be due to low RGL2 accumulation or low endogenous ABA accumulation. However, this is unlikely since we could detect normal protein accumulation of the GA and ABA response factors RGL2 and ABI5, respectively, in *lacs2*, *bdg1*, *gpat4/gpat8* and other cutin biosynthesis mutants under low GA conditions (S10 Fig).

Altogether, these observations strongly suggest that the observed TR events in *lacs2*, *bdg1* and *gpat4/gpat8* mutants under low GA conditions are not due to alterations in GA and ABA signaling pathways.

**Fig 10:**
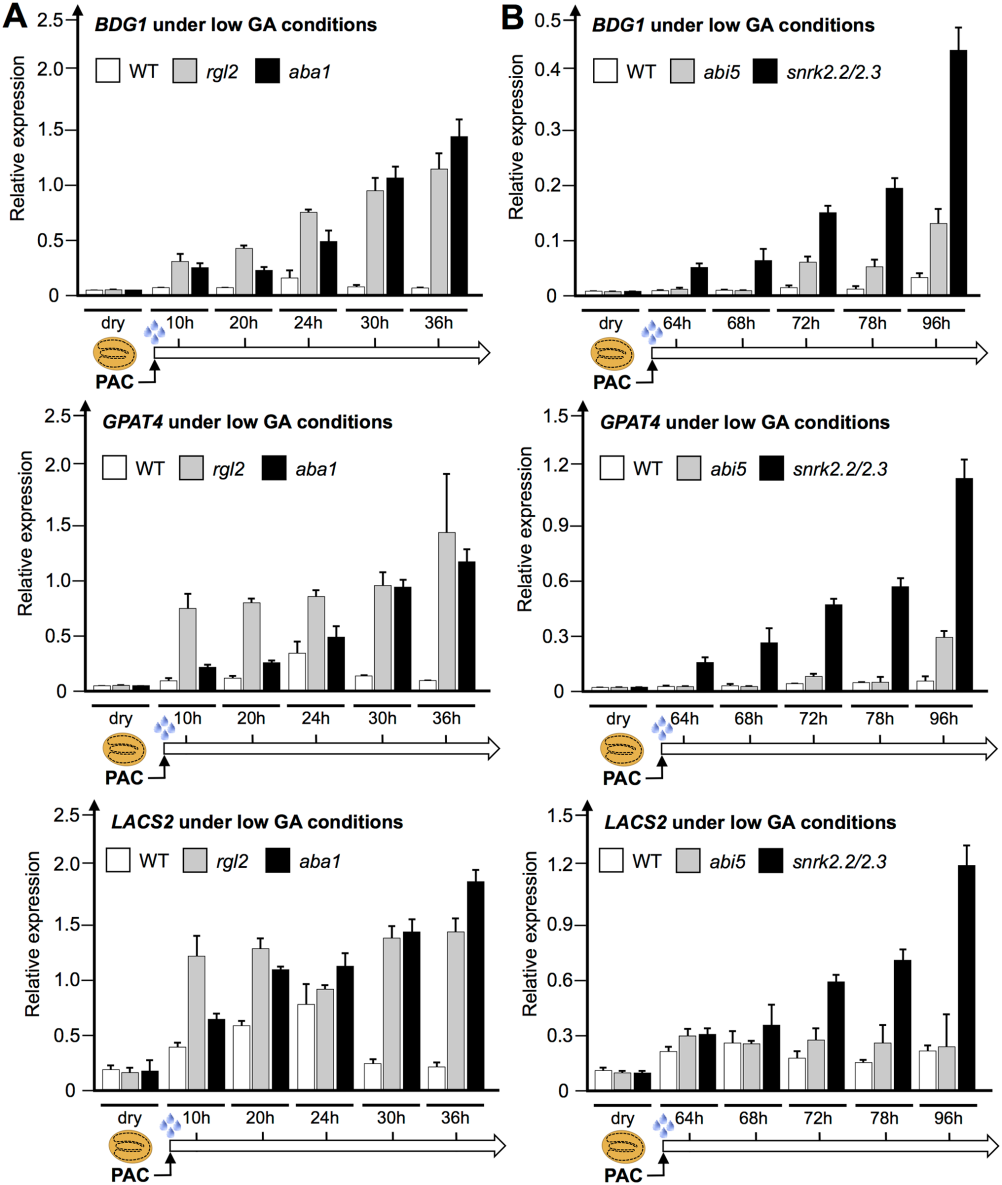
Expression of cutin biosynthetic genes is repressed by ABA under low GA conditions. (A) Histograms show the relative *BDG1*, *GPAT4* and *LACS2* mRNA accumulation in dry seeds as well as upon seed imbibition under low GA conditions in WT (Col), *rgl2* and *aba1*. (B) Same as (A) but with *abi5* and *snrk2*.*2/snrk2*.*3* mutants.

Concerning how GA signaling regulates cutin biosynthesis gene expression, we already showed above that *LACS2*, *BDG1* and *GPAT4* gene expression is repressed when seeds are arrested due to low GA conditions (Fig 4). In contrast, *rgl2* mutants under low GA conditions had higher *LACS2*, *BDG1* and *GPAT4* expression (Fig 10A). This is consistent with the microarray results discussed above. Furthermore, under normal conditions *rgl2* mutants displayed the normal increase of *LACS2*, *BDG1* and *GPAT4* expression upon seed imbibition (S11 Fig). Thus, low GA conditions lead to RGL2-dependent repression of cutin biosynthesis gene expression.

Concerning the role of ABI5, the expression of *BDG1*, *GPAT4* and *LACS2* was strongly repressed in *abi5* mutants under low GA conditions (Fig 10B). However, this repression was not as pronounced as in WT seeds, suggesting that ABI5 together with other ABA response factors repress *BDG1*, *GPAT4* and *LACS2* expression. Indeed, ABI5 is not the only ABA response factor in seeds [52]. Thus, to test the role of ABA we examined the expression of these genes in *aba1-6* mutants, deficient in endogenous ABA synthesis. *BDG1*, *GPAT4* and *LACS2* expression was higher in *aba1-6* mutants under low GA conditions, which was associated with normal RGL2 protein levels (Fig 10A and S12 Fig). Normal *BDG1*, *GPAT4* and *LACS2* expression was observed in *aba1-6* under normal conditions (S11 Fig). Similarly, mutants deficient in *SNF1-RELATED PROTEIN KINASES 2* and *3* (*snrk2-2/snrk2-3*), deficient in ABA signaling, also had higher *BDG1*, *GPAT4* and *LACS2* expression (Fig 10B) [26, 53].

Altogether, these results are consistent with the notion that RGL2-dependent activation of endogenous ABA synthesis and signaling is necessary to repress *LACS2*, *BDG1* and *GPAT4* expression and TR under low GA conditions. They also suggest that cutin depositions in mature seeds play a role to prevent TR under low GA conditions.

## Discussion

### Summary

**Fig 11:**
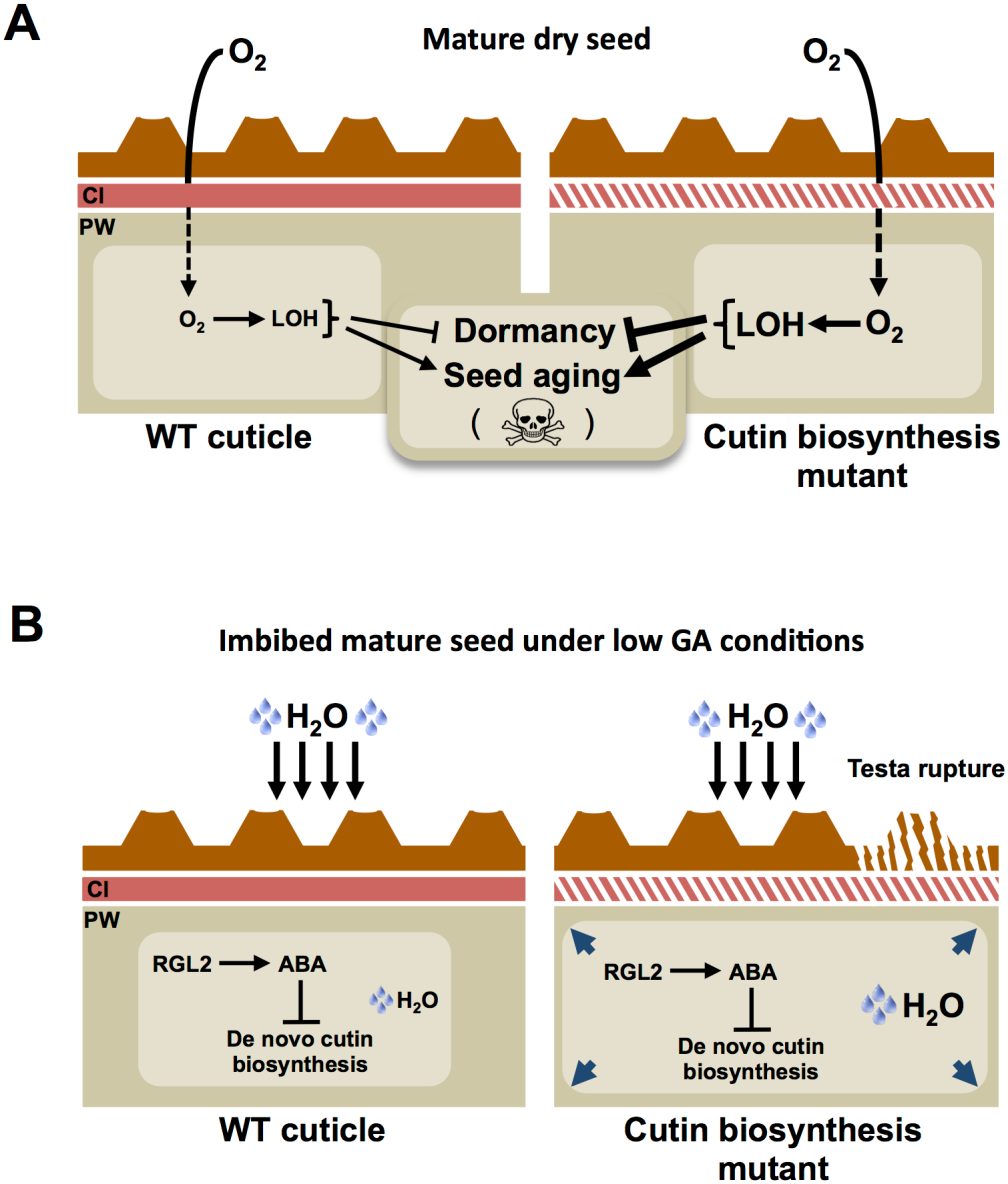
Hypothetical role of a cutin-containing endospermic cuticle in seed physiology. (A) A cuticle limits the diffusion of oxygen within seeds. In cutin biosynthesis mutants, the structure is deficient, which leads to higher diffusion of oxygen and thus higher accumulation of LOH. In turn, higher oxidative stress accelerates loss of dormancy and seed aging. (B) When GA synthesis is blocked, micropylar endosperm cell expansion and testa rupture are blocked. Under these conditions, de novo cutin biosynthesis gene expression is blocked. The cuticular structure already present in dry seeds is therefore maintained and participates to prevent cell expansion and testa rupture. Cell expansion involves water uptake within cells. The role assigned to the endospermic cuticle in this context is suggested by the fact that cutin biosynthesis mutants fail to repress cell expansion and testa rupture. Cl: cuticle, PW: primary cell wall.

We used whole-genome approaches to identify common GA and ABA signaling gene expression programs. This led us to identify cutin biosynthesis gene expression being markedly repressed when seeds are arrested under low GA conditions (Figs 1 and 4). Although this repression does not require a functional *ABI5* gene, perhaps due to redundancy with other similar TFs, it does nevertheless necessitate endogenous ABA biosynthesis and signaling (Fig 10). This result was initially perplexing given the osmotolerant state of arrested seeds and the known participation of cutin to prevent transpiration in leaves. Researchers previously identified cutin-containing layers and showed their role in regulating the seed’s permeability [31–34]. Here we showed that cutin biosynthesis genes play fundamental roles in seed physiology by regulating 1) endosperm permeability as shown in the context of *bdg1* mutants, 2) seed viability, 3) seed dormancy and 4) repression of TR (Figs 3 and 5–8). Furthermore, histological analysis identified in mature seeds an electron-dense endospermic cuticular film covering endosperm cells on their external side (Fig 2). The endospermic cuticle most likely contains cutin or cutin-like depositions since its morphology is altered in *bdg1* mutants. This layer is therefore strategically positioned to influence water and gaseous exchanges between the outer environment and the seed’s living tissues. This view is supported by the observation that 1) the endosperm can markedly restrict the penetrance of the dye towards the embryo and 2) *bdg1* mutant endosperm is more permeable to toluidine blue (Fig 6). In contrast, coat-less WT and *bdg1* embryos are highly and similarly susceptible to staining by the dye, suggesting that they are not significantly shielded from the outer environment and consistent with the absence of a thick embryonic cuticle (S4 Fig). Furthermore, consistent with the notion that cutin or cutin-like depositions are necessary to shield the seed against oxidative damage, quantitative measurements showed that *bdg1*, *lacs2* and *gpat4*/*gpat8* mutants accumulate higher levels of esterified fatty acid hydroxides (LOHs), which leads to decreased tocopherol levels (Fig 9). Fig 11 compiles the main findings of this work. We further discuss each of these findings in turn.

### Endospermic cuticle and TR

Under standard germination conditions, initial seed imbibition leads to a passive and rapid water uptake lasting 60 minutes. Thereafter a period of about 30 hours ensues where water content in seeds plateaus, or slowly rises, before it increases again to accompany the cellular expansion needed to allow the embryo to germinate. TR normally occurs about 24 hours upon seed imbibition [45, 46]. TR is not a fully understood process. However, authors proposed that TR is driven by micropylar endosperm cell expansion, itself driven by active cellular water uptake and primary wall modifications that could include pectin methylesterase activities [46, 47]. Not surprisingly, genes encoding methylesterases as well as other cell wall modifying enzymes were up-regulated in *rgl2* mutant seeds that are able to rupture testa under low GA conditions (S1 Fig). According to this view, absence of TR in WT seeds under low GA conditions should be associated with lack of micropylar cell expansion. Here we could show that this is indeed the case (Fig 5). Furthermore, we showed that *lacs2* and *bdg1* mutants fail to repress TR when GA synthesis is blocked. This correlated with their inability to block not only the expansion of the micropylar endosperm but also that of the peripheral endosperm, although to a lower extent (Fig 5). As expected, cell expansion was accompanied by an increase in cellular transparency, consistent with the notion that it involves water uptake (see model in Fig 11). The presence of a cuticular layer surrounding the endosperm suggests that it could directly be involved in preventing endospermic cellular expansion in response to low GA conditions. Since it is not well understood how GA promotes micropylar endosperm expansion, the nature of the mechanisms involved remains unclear. The endospermic cuticle rigidity or permeability could directly influence cell wall plasticity or the endosperm’s water relations, respectively, in the context of control of cellular expansion. How exactly it does so is unclear. Perhaps, the fact that not all cutin biosynthesis gene mutations lead to high percentages of TR in presence of PAC, as in *dcr*, *lcr* or *gpat4*/*8* mutants (Fig 3), could indicate that it is a matter of rigidity. Indeed, our data indicate that a majority of cutin biosynthesis mutants studied had seed viability and dormancy phenotypes. These phenotypes are best explained as a result of higher permeability to outer molecules.

### Control of TR by GA and ABA signaling pathways

We previously showed that RGL2 is necessary to repress TR under low GA conditions. We also showed that absence of seed germination under low GA conditions requires RGL2-dependent stimulation of endogenous ABA accumulation and ABI5 activity [26]. Here we could show that endogenous ABA synthesis is also required to repress TR under low GA conditions (S9 Fig). In contrast, exogenous ABA does not efficiently repress TR [49]. Thus it appears that ABA does play a role to repress TR but only in the context of high RGL2 accumulation triggered by low GA conditions.

Interestingly, it is also under low GA conditions that the most marked repression of cutin biosynthesis gene expression could be observed. Indeed, exogenous ABA only delayed the onset of *LACS2*, *BDG1* and *GPAT4* expression in imbibed seeds, which coincided with the onset of the first visible TR events (S13 Fig). We speculate that failure to efficiently repress cutin biosynthesis could explain the occurrence of TR in presence of exogenous ABA [49]. Indeed, leaky expression of cutin biosynthesis genes in presence of exogenous ABA could lead to modification of pre-existing cutin-like depositions in dry seeds. This in turn could affect control of micropylar endosperm cell expansion. In favor of this view, previous reports have shown that *aba* mutants have an increased cuticular permeability [54]. Furthermore, we found that under normal germination conditions, induction of cutin biosynthesis gene expression takes place in both endosperm and embryo (S14 Fig). Cutin biosynthesis gene expression in the endosperm is consistent with the notion that it may participate to modify pre-existing cutin-like depositions in endosperm cells. Thus efficient blockade of TR would require efficient blockade of de novo cutin biosynthesis, as under low GA conditions, in order to maintain intact endospermic cuticular structures typical of mature seeds. However, previous reports have shown that Arabidopsis seed germination can proceed, although in a much-delayed manner, even in presence of transcription inhibitors [55]. Therefore, it is possible that de novo cutin biosynthesis gene transcription would facilitate rather than play an essential role for testa rupture.

### Cuticular structures in Arabidopsis mature seeds

The occurrence of cuticular structures in mature seeds has not been extensively explored. However, more information is available during embryogenesis. Beeckman et al identified an electron dense layer, likely containing lipids, bordering the inner integument 1 (ii1) on the surface facing the one-celled zygote and, at later stages of embryogenesis, the immature endosperm [56]. The epidermal origin of integuments led Beeckman et al to consider this layer as the original cuticle of ii1 [56, 57]. Consistent with this view, Molina et al detected *CYP86A2* promoter activity specifically in ii1 cells. Furthermore, this cuticle is found only on the surface of cells in direct contact with the embryo sac and not in the other integument layers [56]. Although this layer was visible until the mature embryo stage, its visualization in mature seeds was not confirmed [32, 56]. *CYP86A2* promoter activity also decreases during seed maturation and its seed coat localization becomes difficult due to the natural collapse of the integuments [32]. Together with lipid polyester measurements in dry seeds, Molina et al. concluded that a cutin-like polyester layer is likely associated with the inner seed coat in mature seeds [32].

Our histological analysis confirms the existence of a cuticle located between the inner seed coat and the endosperm layer. Remarkably, this layer appears to be tightly associated with the outer endospermic primary wall (PW) rather than the testa (Fig 2 and S3 Fig). This could suggest an endospermic origin. However, previous work showed that during the early-heart stages of embryogenesis growth of cell wall material is initiated from the inner integuments thus providing the cell wall material required for endosperm cellularization [58]. Thus, it seems plausible that the endosperm-associated cuticle observed here originated from ii1 cells during embryogenesis. The latter hypothesis may be supported by publicly available gene expression localization data during seed development. Indeed, during the heart stage and thereafter, *BDG1*, *LACS2* and *GPAT4* are more highly expressed in seed coat maternal tissues than in the endosperm or the embryo (S15 Fig). However, more complex scenarios cannot be excluded, such as the maternal integuments and endosperm cells both being responsible for the manufacture of the cuticle in an unforeseen complex manner.

Concerning the embryo, previous reports have provided evidence that the embryo is surrounded by a cuticle layer during embryogenesis [32, 59]. The *ZHOUPI* gene encodes a basic Helix-Loop-Helix TF expressed in the endosperm during embryogenesis [59]. Mutants in this gene (*zou* mutants), have a thicker and disorganized cuticle layer covering the cotyledons that is surprisingly similar to that observed in *bdg1* mutant leaves [10]. Furthermore, *zou* mutants are unable to breakdown the endosperm, which also abnormally adheres to the embryo during seed development, and have altered seed morphology [10, 59]. In mature seeds, we could not observe a clear embryonic cuticle or at least any structure comparable in thickness to the cuticle observed in the outer PW of endosperm cells (S4 Fig). Furthermore, we did not observe significant seed morphological defects in cutin biosynthesis mutants nor endosperm-embryo adherence problems during seed germination. However, ZHOUPI being a TF, it could affect cutin-like biosynthesis and deposition more severely than single cutin biosynthetic gene mutations. Thus cutin biosynthesis genes could indeed still be important for proper seed development.

### Physiological role of cutin biosynthesis: seed viability and dormancy

A significant finding of this work is that cutin biosynthesis mutant seeds are less viable and less dormant. Intriguingly, the endosperm cuticle is markedly thicker relative to that found in leaves (10 fold thicker). This could be consistent with a role to limit exchanges with outer molecules, as directly suggested with the toluidine blue dye experiments (Fig 6), including gaseous exchanges with the seed’s outer environment. Indeed, previous reports have shown that *lacs2* and *bdg1* mutants produce higher amount of reactive oxygen species (ROS), most likely as a result of a more permeable cuticle [54]. Furthermore, mutant seeds over-accumulating reactive oxygen species (ROS), such as *cat2-1* and *vet1-1*, deficient in H_2_O_2_^-^ dismutating catalase (CAT) activity and tocopherol cyclase activity, respectively, display low dormancy [20]. In contrast, a *rboHD* mutant, deficient in NADPH oxidase, catalyzing the production of apoplastic superoxide from oxygen and NADPH, is highly dormant [20]. The *vet1* mutant is also known to display lower seed viability [60]. Here, we found that cutin biosynthesis mutant seeds accumulate higher lipid oxidation species, which leads to lower tocopherol levels (Fig 9). We therefore propose that lower viability and dormancy in cutin-biosynthesis mutant seeds could be the result of enhanced oxidative stress as a result of a deficient endospermic cuticle in mature dry seeds [61].

Previous work identified *HISTONE MONOOUBIQUITINATION1* (*HUB1*) and its homolog *HUB2*, as positive regulators of seed dormancy [62]. HUB1 and HUB2 are C3HC4 RING finger proteins involved in the monoubitination of histone H2B. How HUB1 and HUB2 promote dormancy remains challenging, particularly considering that *hub1* and *hub2* are known to display pleiotropic phenotypes [63]. Interestingly, *hub1* and *hub2* mutants were recently found to display disorganized cuticle layers in leaves, which was associated with increased transpiration rates [64]. These phenotypes were proposed to result from specific activation of cuticle biosynthesis genes, including that of *LACS2*. Thus, our findings indicate that a possible explanation for the low dormancy of *hub1* and *hub2* mutant seeds resides in a deficient cuticle in seeds.

### Perspectives and speculations

Our identification of an electron dense, cuticular structure covering the endosperm on its external side poses the question of its occurrence and physiological role in seeds of other angiosperms. Given our evidence that cutin biosynthesis genes protect the seed against oxidative damage, we could expect that similar cuticular structures will be co-opted in a wide range of seed plants [35, 65–67]. This expectation is further strengthened considering the likely role of oxidation in regulating seed dormancy in other species [68]. In what regards gymnosperms, the embryo is not surrounded by an endosperm. It would therefore be interesting to examine the occurrence of cuticles in gymnosperm seeds, including embryonic cuticular layers.

## Materials and Methods

### Plant material

The *Arabidopsis thaliana* mutant *rgl2-13* was isolated by Tyler et al. [69] and obtained from T.P. Sun. The mutant *aba1-6* was isolated by Niyogi et al. [70].

The *abi5-3* mutant was first described by Finkelstein and Lynch [27]. The double mutant *snrk2-2/snrk2-3* was obtained from J.K. Zhu. Cutin biosynthesis mutant *lacs2-3* was isolated by Bessire et al. [71], *bdg1* by Kurdyukov et al. [10], *lcr* by Wellesen et al. [42], *dcr* by Rani et al. [72], *gpat4/8* by Li et al. [7], *gpat6-1* and *gpat6-2* by Yang et al. [40] and the suberin biosynthesis mutant *gpat5* by Beisson et al. [36]. They were obtained from Dr. Christiane Nawrath.

### Plant growth conditions and germination assays

All seed batches compared in this study were harvested on the same day from plants grown side by side (i.e., under identical environmental conditions). Dry siliques were obtained around 8 weeks after planting.

Germination assays were performed with two independent seed batches. A minimum of three technical replicates of one batch was plated to measure percentage of testa rupture and endosperm rupture events (standard errors). For germination test, seeds were sterilized with a seeds sterilization solution (1/3 bleach, 2/3 water, 0.05% Tween) and plated on a Murashige and Skoog medium containing 0.8% (w/v) Bacto-Agar (Applichem). ABA conditions and low-GA conditions, respectively, mean that ABA (5 μM) and PAC (5 μM), an inhibitor of GA synthesis, were added to the medium. Plates were incubated at 21–23°C, 16 h of light per day, light intensity of 80 μE·m−2·s−1, humidity of 70%). Between 100 and 200 seeds were examined with a Stemi 2000 (Zeiss) stereomicroscope and photographed with a high-resolution digital camera (Axiocam zeiss) at different times of seed imbibition. Photographs were enlarged electronically for measurement of testa and endosperm rupture events.

### Dormancy test

Dormancy assays were performed with two independent seed batches. Seeds from each batch were harvested at the same day and three replicates of 100–200 seeds were plated to measure percentage of testa and endosperm rupture events (standard errors). Freshly harvested seeds were stored at room temperature for 2 weeks and 2 months (after ripening) to be used as after-ripened material. Seeds were sterilized and plated on MS medium. The seeds were immediately irradiated with a far-red pulse of 5 minutes (FR, 4.5 μmol·m−2·sec−1) followed by a red light pulse of 5 minutes (R, 20 μmol·m−2·sec−1). Plates were wrapped in several layers of aluminum foil and seeds were germinating in darkness. Dormancy assays in darkness (S7 Fig) omitted the FR and R pulses.

### Accelerated aging and seed viability test

Dry seeds were exposed to an accelerated aging procedure by storing them in a high humidity (HR 85%) and high temperature (40°C) conditions during 2, 4 or 6 days [17].

Germination rates were measured by plating 50–100 seeds in Petri dishes containing a filter paper on the top of cotton wool imbibed with deionized water. The seeds were not sterilized and were germinating at 15°C in darkness. Experiment was repeated with two independent seed batches harvested at the same time. Three replicates of each batch were plated to measure percentage of endosperm rupture (standard errors).

### ChIP-Seq assay

Described in Lee et al. [39].

### Microarray

Total RNA was isolated from WT (Col) and *rgl2-13* seeds imbibed in presence on PAC (5 μM) for 24 and 36 hours. 400 ng of total RNA were used to synthesize biotinylated cRNA using MessageAmp III kit (Ambion, Thermo Fisher Sientific) according to the manufacturer’s recommendations. 10 μg of amplified targets were hybridized on Affymetrix GeneChip Arabidopsis ATH1 Genome arrays. Detailed analysis of microarray data is provided in S1 Table. Mapman software was used to extract a list of genes related to cell wall and cuticle formation [38]. Clustering of selected genes was performed using Gene Cluster 3.0 software using the Euclidean distance method and average linkage. The pictures were generated using TreeView.

### RNA extraction and RT-qPCR

Total RNA was extract from 1000–1200 seeds as described by Vicient and Delseny (1999). Total RNAs were treated with RQ1 RNase-Free DNase (Promega) and reverse-transcribed by using Improm II reverse transcriptase (Promega) and oligo(dT)15 primer (Promega) according to the manufacturer's recommendations. Quantitative RT–PCR was performed by using the ABI 7900HT fast real-time PCR system (Applied Biosystems) and Power SYBR Green PCR master mix (Applied Biosystems). Relative transcript level was calculated by using the comparative ΔCt method and normalized to the *PP2A* (*At1g69960*) gene transcript levels. The primers used in this study were designed by Primer Express software for real-time PCR, version 3.0 (Applied Biosystems):

LACS2: 5’ TCCAACAGCCCACAGACAAC

5’ GATGATAAGAACCCATGAATGAACAC

BDG1: 5’ GTCCAACAGCCCACAGACAA

5’ ATGATGATAAGAACCCATGAATGAAC

GPAT4: 5’ ACCTCCGATCACGATTTCATGT

5’ CTGATTTGGTCTCATGCACCAT

### Protein gel blot

Seeds proteins were extracted from around 800 seeds and resolved under reducing conditions using 10% SDS/polyacrylamide gels. Proteins were transferred onto polyvinylidene difluoride membranes (Immobilon-P; Millipore), which were incubated with primary affinity-purified ABI5 antibody (diluted 1:3000; 60 μg/mL) for 4 h at room temperature and with affinity-purified RGL2 antibody (diluted 1:1500; 60 μg/mL) over night h at 4°C. After a first wash of membranes (5% milk in Tris-buffered saline [TBS] + 0.2% Tween for anti-RGL2 and TBS + 0.05% Tween for anti-ABI5), the secondary antibody, peroxidase-conjugated anti-rabbit antibody (Amersham Pharmacia; diluted 1:10,000), was added and membranes were incubated for 1 h at room temperature in TBS supplemented with 5% milk. After incubation, membrane was washed twice (15 min each) with TBS containing 0.05% Tween 20 for anti-ABI5. For anti-RGL2, membrane was washed three times (10 min each) with 5% milk in TBS + 0.2% Tween follow by two washes with TBS + 0.2% Tween (10 min each). After the final wash, immune complexes were detected on x-ray film (Fuji medical x-ray film) in its linear range using the ECL kit according to the manufacturer's specifications (Amersham Pharmacia).

### Histological studies and endospermic cells sizes quantification

Imbibed seeds were fixed overnight at 4°C in phosphate buffer pH 7.2 complemented with 4% formaldehyde and 0.25% glutaraldehyde. They were then washed in phosphate buffer, in distilled water and embedded in a gel of agarose 1.5%. They were then dehydrated using a graded ethanol series, cleared in Neoclear and embedded in paraffin. 12 μm-thick sections were cut with a microtome, placed on SuperFrost slides (Roth), deparaffinized with Neoclear and rehydrated in a graded ethanol series. Samples were stained with Sudan red 7B 0.1% in a 1:1 mix of polyethylene glycol 400 and glycerol 90% during 2 min (Fig 2A and 2B) or 30 min (Fig 5) and finally mounted in glycerol. Imaging was done using an Eclipse 80i widefield microscope (Nikon) equipped with a 40x Plan Fluor NA 0.75 lens, DIC optics and a Digital Sight DS-Fi1 color camera. Image analysis and quantification were performed using ImageJ (1.49i) (W.S. Rasband, http://imagej.nih.gov/ij/). Number of seeds used: WT (MS), 35(1h) and 33(24h); WT (PAC), 33(1h) and 21(120h); *lacs2* (MS), 37(1h) and 30(24h); *lacs2* (PAC), 37(1h) and 42(120h); *bdg1* (MS), 28(1h) and 33(24h); *bdg1* (PAC), 28(1h) and 43(120h).

### Electron microscopy

Imbibed arrested seeds (PAC-treated) and mature leaves (1 month-old Col-0 plants grown under short days condition) were fixed overnight at 4°C under agitation in 2.5% glutaraldehyde in 100mM cacodylate sodium buffer pH 7.0 complemented with 0.01% Tween-20. To improve the fixation and subsequent resin infiltration, seeds were delicately punctured with a fine needle 1h30 after the start of fixation. After fixation they were washed in cacodylate sodium buffer, post-fixed in 1.5% OsO4 for 2h at 4°C, washed in cacodylate buffer and in milliQ water, and finally post-fixed in1% aqueous uranyl acetate during 1h at 4°C. Seeds were then embedded in a gel of agarose 1.5%, dehydrated in a graded ethanol series and embedded in Epon 812. Ultrathin cross sections (85 nm) were cut and stained with 2.5% uranyl acetate and Reynolds lead citrate. The sections were viewed in a FEI Tecnai G2 Sphera transmission electron microscope at 120 kV.

Image treatment and analysis was done using Fiji [73]. Cuticle and primary wall widths were measured along lines perpendicular to the direction of the cell wall. The measurements were done on 39 electron micrographs of Col-0 seeds, representing at least 24 cells and 5 seeds, and 9 electron micrographs of Col-0 leaves from 9 cells. For each image, the cuticle and the primary wall widths were estimated from the average of two measures done at two different positions.

Cuticle density was also measured along lines perpendicular to the direction of the cell wall, starting at the end of the primary wall and ending at the cuticle end. 36 and 33 electron micrographs were analysed for Col-0 and *bdg1* seeds respectively. To maximize the cuticular area analysed while minimizing any bias regarding the choice of the regions analysed, measurements were done on lines systematically drawn along the cell walls with a regular spacing of 200nm; 969 and 1002 lines were measured in Col-0 and *bdg1* seeds respectively, representing at least 23 and 22 cells in 5 and 6 seeds respectively. To reduce noise, a Gaussian blur of radius 1.5 pixel was applied on each image before measuring the cuticle density. The intensity profile along each line was measured, as well as the extracellular background intensity (which was measured on a neighbouring area on the same image). The optical density at each point of the line was defined as the logarithm (in base 10) of the ratio of the extracellular background divided by the intensity of the pixel at the position considered. To be able to average all lines, each profile was scaled between 0 and 100, the position 0 representing the primary wall/cuticle interface, and the position 100 the end of the cuticle (see Fig 2H–2I). A spline interpolation was subsequently performed on each line profile using Matlab (MathWorks) in order to re-sample each profile between 0 and 100 with a sampling interval of 1. Averaging between all profiles of a given genotype was then performed, and plotted +/- standard errors of the mean.

### Toluidine blue staining

Seeds were plated on MS medium supplemented with ABA (3 μM) or PAC (5 μM). For MS conditions WT and *bdg1* seeds were harvested 12 h after imbibition when no testa rupture events could be observed. Seeds were incubated at room temperature with toluidine blue (0.05%) for 6 h in microcentrifuge tubes and afterwards dissected to observe embryos coloration. For ABA conditions seeds were harvested 4 h (when no testa rupture events occurred) and 36 h after imbibition (when testa rupture occurred). Seeds were incubated at room temperature with toluidine blue (0.05%) supplemented with ABA to prevent endosperm rupture. For PAC conditions seeds were harvested 36 h after imbibition. At this time no testa rupture events occurred. Seeds were incubated at room temperature with toluidine blue (0.05%) supplemented with PAC (5 μM) to keep seeds arrested. For embryo staining, seeds were plated on ABA and harvested 4 h after imbibition. Seeds were dissected and embryos were incubated for 10 min in toluidine blue (0.0125%)(1:4 dilution).

### Statistics

The comparison of cuticle densities (Fig 2L) was done between corresponding points of the average cuticle density profiles of the Col-0 and the *bdg1* phenotypes using Kolmogorov-Smirnov tests under Matlab (MathWorks). Statistical analyses on Fig 5A and 5B were performed using R (http://CRAN.R-project.org). p-values correspond to Student’s t test, which has been performed after the normality of the distributions studied was verified using Kolmogorov-Smirnov tests.

### Linoleic and linolenic hydroxides analysis

18:2 and 18:3 hydroxides were quantified by normal and reverse HPLC as previously described with minor modifications using 15 mg of dry seeds as starting material [60]. 18:2 and 18:3 hydroxides were first purified by RP-HPLC from saponified lipid extracts. Fractions were evaporated and LOHs were separated and quantified by NP-HPLC using authentic 9(S)- and 13(S)-hydroxy octadecadi(tri)enoate (Cayman chemical, MI, USA). Unmodified 18:2 and 18:3 were quantified as FAME by GC-FID (HP7890, Agilent Technologies, CA, USA) equipped with a 25m x 0.25 mm DB-23 capillary column. Glycerol triheptadecanoate (Sigma) was used as an FA internal standard.

### Tocochromanol analysis

Tocochromanols were analyzed by NP-FLD-HPLC as previously described with minor modifications [50]. Total lipids were extracted 3 times from 5 mg of dry mature seed in presence of BHT (antioxidant) and 2 μg of tocol (internal standard). Extracts were separated by isocratic HPLC (HP1260 Infinity, Agilent Technologies, Palo Alto, CA) on a diol column (LiChrospher-100 Diol 250 x 4 mm, Merck) using hexane/*tert* butyl methyl ether (96/4, v/v) as a mobile phase at 1.5 mL/min. Tocochromanols were detected with a FLD detector set at l_ex_ = 295 nm and l_em_ = 330 nm. Quantifications were performed using 6-point calibration curves directly built on the instrument software with authentic tocochromanol standards normalized with the internal standard (Matreya, PA, USA). Regression coefficients of standard curves are equal to 0.995 for the γ-tocopherol, to 0.997 for α-tocopherol and 0.999 for α-tocopherol.

## Supporting Information

S1 Fig Expression of genes involved in cell wall formation is potentially regulated by RGL2. (A) Dynamics of cell wall formation gene expression between *rgl2* and WT seeds is represented with a color code. The scale bar relates color with absolute fold changes.
(TIFF)

S2 Fig Staining of the cuticle of leaves epidermal cells. Sudan Red stains the cuticle of epidermal cells of mature leaves, as indicated by the pink line delineating the outer wall of these cells (arrows). Bar: 10μm.
(TIFF)

S3 Fig Examples of the cuticle of Col-0 and *bdg1* endosperm cells. Close-ups of the outer side of endosperm cells in Col-0 seeds (A) and *bdg1* seeds (B). Each electron micrograph represents a different cell, and a variety of cuticular defects are visible in *bdg1* cells (B). Note that during the preparation of the specimens for electron microscopy, the testa sometimes gets detached from the endosperm and is sometimes not visible in proximity of the endosperm cell wall (A, right image; B, bottom middle image). Abbreviations are: T: testa; C: cuticle; PW: primary wall. Bars: 300nm.
(TIFF)

S4 Fig Comparison of endospermic and embryonic cell walls. (A) Electron micrograph of a Col-0 endosperm cell (bottom left) and a neighboring embryonic cell (top right) having a potential cuticle. The inset in the bottom right corner shows, with the same magnification, the primary cell wall and the cuticle observed on the outer side of an endosperm cell in the same seed and on the same electron microscopy grid. Bar: 1000nm. (B) Close-up of the boxed area in (A). The inset in the bottom left corner shows a close-up of the boxed area in the inset in (A). Bar: 300nm.
(TIFF)

S5 Fig Cutin biosynthesis mutants germinate normally under standard germination conditions and can also germinate under low GA conditions. (A) Images showing WT (Col), *lacs2*, *lcr*, *bdg1*, *dcr*, *gpat4/8*, *gpat5* seeds 36h after imbibition under normal germination conditions. Chart indicates percentage of testa rupture over time under normal germination conditions (two independent batches (*n* = 150–200)). (B) Images showing WT (Col), *lacs2*, *lcr*, *bdg1*, *dcr*, *gpat4/8*, *gpat5* seeds 7days after imbibition under low GA conditions. Chart indicates percentage of endosperm rupture over time under low GA conditions (two independent batches (*n* = 150–200)).
(TIFF)

S6 Fig Toluidine blue dye is localized on the outer side of the testa while leaving the internal side, with the endosperm still attached, colorless. Image showing dissected WT seed coat (testa + endosperm) from PAC-treated seeds after 6 hours of toluidine blue incubation as in Fig 6. Yellow arrow indicates inner side of the seed coat (that includes the endosperm). Black arrow indicates the outer side of the seed coat.
(TIFF)

S7 Fig Freshly harvested *lacs2*, *bdg1*, and *gpat4*/*8* mutants exhibit low dormancy in darkness. (A) WT (Col), *lacs2*, *bdg1*, *gpat4/8* seeds seed germination in darkness was assessed 3 days after imbibition. (B) Histograms represent the percentage of germination of WT (Col), *lacs2*, *bdg1* and *gpat4/8* seeds 3 days after imbibition in darkness (For each independent seed batches seeds were plated in duplicate (*n* = 100–150)).
(TIFF)

S8 Fig *vte2/vte1* double mutant seeds, unable to synthesize the antioxidant tocopherol, accumulate high LOH levels. (TIFF)

S9 Fig PAC-treated *aba1-6* mutants, unable to synthesize ABA, display TR. (A) Images showing WT (Col), *aba1 and rgl2* seeds 36h after imbibition under low GA conditions. (B) Chart represents percentages of testa rupture over time (in hours) under low GA conditions (3 replicates (*n* = 50–100)).
(TIFF)

S10 Fig Imbibed seeds of cutin biosynthesis mutants accumulate high levels of RGL2 and ABI5 protein under low GA conditions. Protein gel blot analysis of RGL2 and ABI5 proteins. Ten micrograms of total proteins extracted from seeds harvested at 72 h after imbibition in presence of PAC was used per lane. Protein extracts were stained with Ponceau S as a loading control prior to detection with antibodies against RGL2 and ABI5.
(TIFF)

S11 Fig Expression of cutin biosynthetic genes in *rgl2* and *aba1* mutants is normal under normal germination conditions. Histograms show the relative *BDG1*, *GPAT4* and *LACS2* mRNA accumulation in dry seeds and upon seed imbibition under normal germination conditions in WT (Col) as well as in *rgl2* and *aba1* mutants.
(TIFF)

S12 Fig RGL2 protein level is high in *aba1* under low GA conditions. Protein gel blot analysis of RGL2 protein. Ten micrograms of total protein was used per lane. Protein extracts were stained with Ponceau S as a loading control prior to detection with antibodies against RGL2. Percentage of testa rupture is indicated under each time point.
(TIFF)

S13 Fig Expression of cutin biosynthetic genes is weakly repressed by exogenous ABA. Histograms show the relative *BDG1*, *GPAT4* and *LACS2* mRNA accumulation in WT (Col) dry seeds and upon seed imbibition in absence (MS) and presence (ABA) of exogenous ABA. Expression of these genes coincides with the testa rupture events.
(TIFF)

S14 Fig Expression of cutin biosynthetic genes is induced in both endosperm and embryo during early seed germination. Histograms show the relative *BDG1*, *GPAT4* and *LACS2* mRNA accumulation in both endosperm and embryo of WT (Col) seeds imbibed under normal germination conditions. The expression in dry seed (dry) is also shown.
(TIFF)

S15 Fig Expression of cutin biosynthetic genes during embryogenesis. Data shown were retrieved from the Arabidopsis eFP browser website (http://bar.utoronto.ca/efp/cgi-bin/efpWeb.cgi). Original data published in Le et al 2010. Winter et al. PLoS One. 2007 Aug 8;2(8):e718; Le et al PNAS 2010 May 4;107(18):8063–70
(TIFF)

S1 Table Transcriptome analysis WT vs *rgl2*. (XLS)

S2 Table HA-ABI5 ChIP-Seq analysis. (XLS)

S1 File Microarray report. (PDF)
