# Supplementary figures and images for: An Endosperm-Associated Cuticle Is Required for Arabidopsis Seed Viability, Dormancy and Early Control of Germination
Source: PLoS Genet. 2015 Dec 17;11(12):e1005708. doi: 10.1371/journal.pgen.1005708 (PMC4683086; doi:10.1371/journal.pgen.1005708)

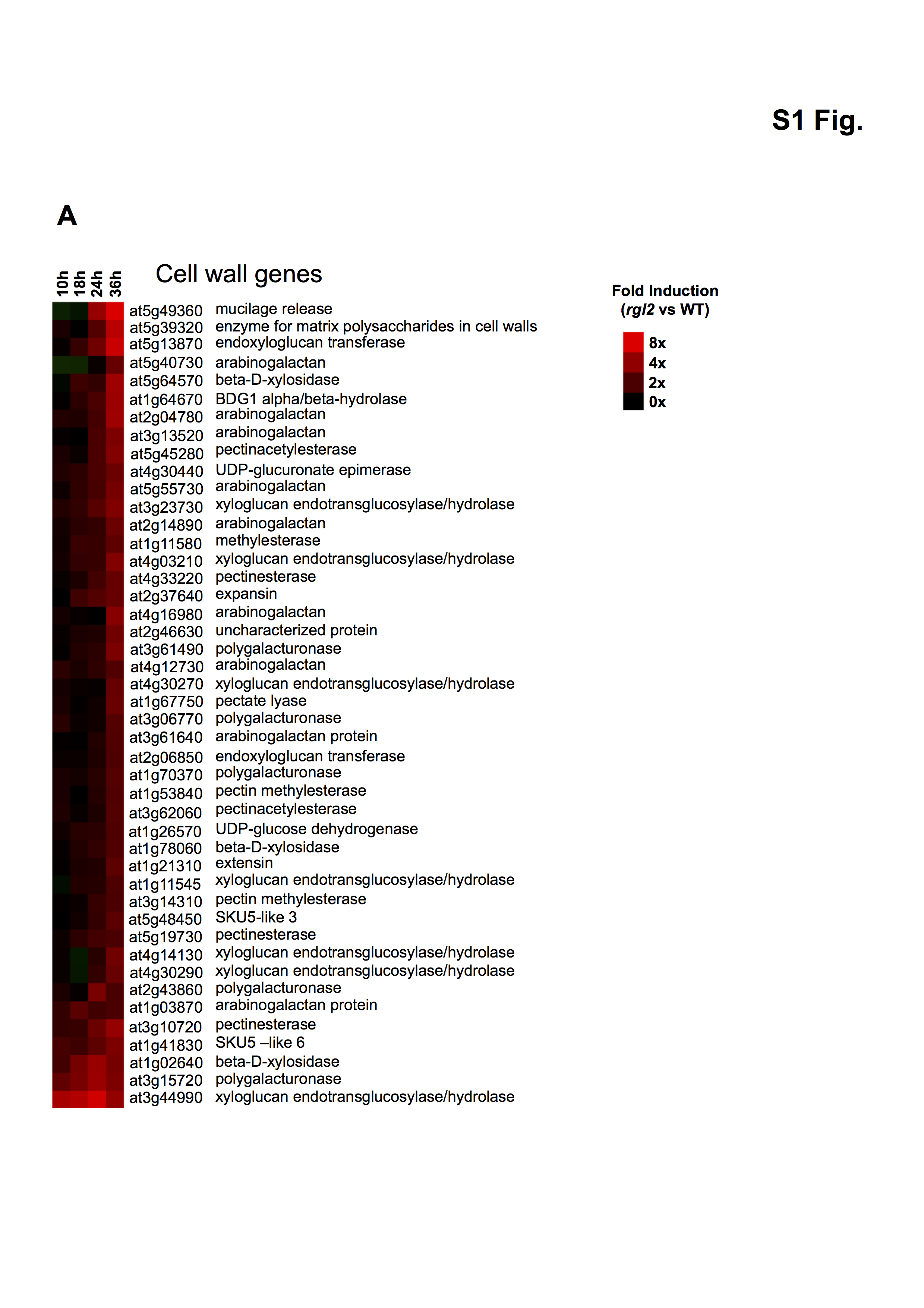

Supplement: S1 Fig — (A) Dynamics of cell wall formation gene expression between rgl2 and WT seeds is represented with a color code. The scale bar relates color with absolute fold changes. (TIFF) [file pgen.1005708.s001.tiff]

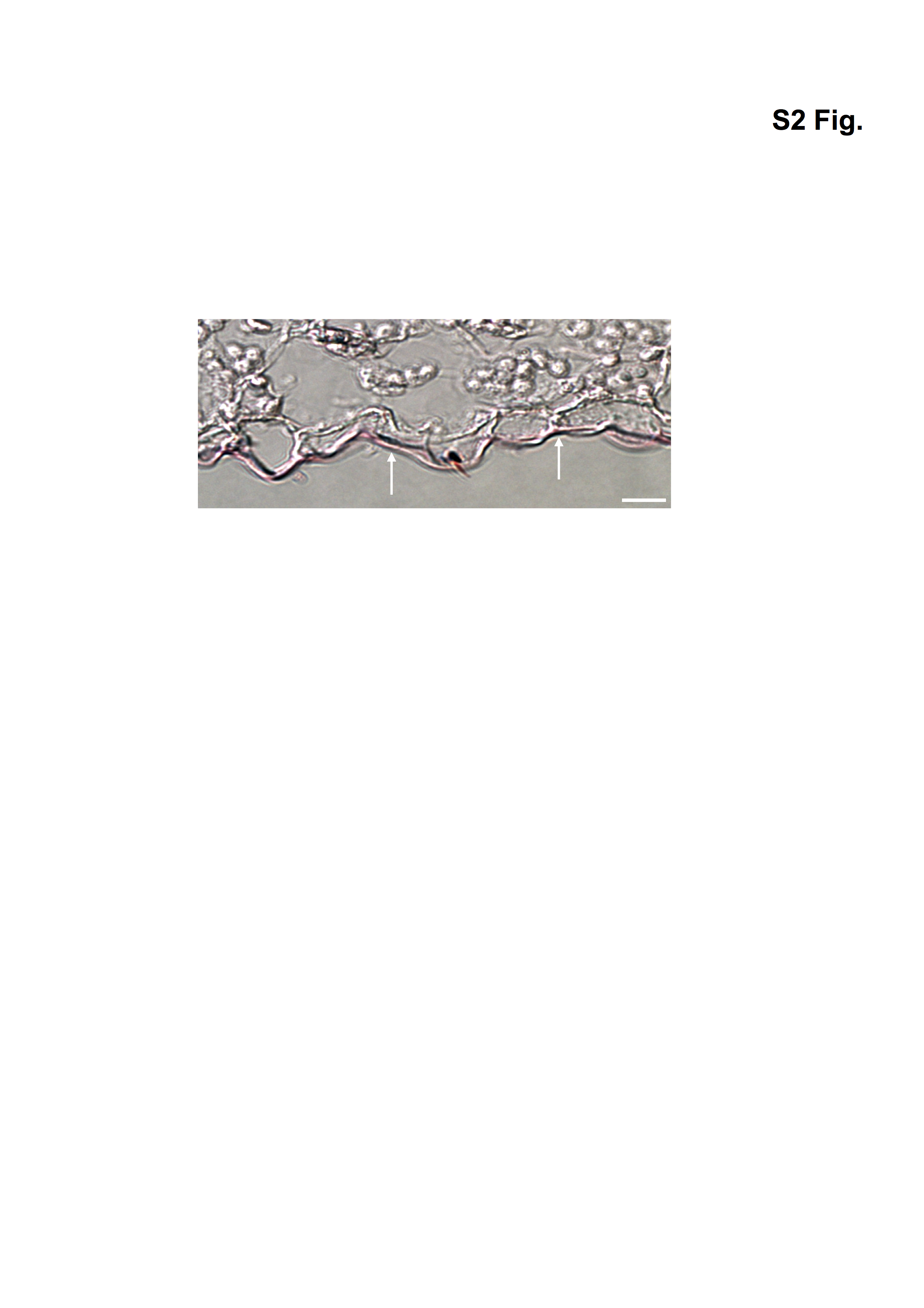

Supplement: S2 Fig — Sudan Red stains the cuticle of epidermal cells of mature leaves, as indicated by the pink line delineating the outer wall of these cells (arrows). Bar: 10μm. (TIFF) [file pgen.1005708.s002.tiff]

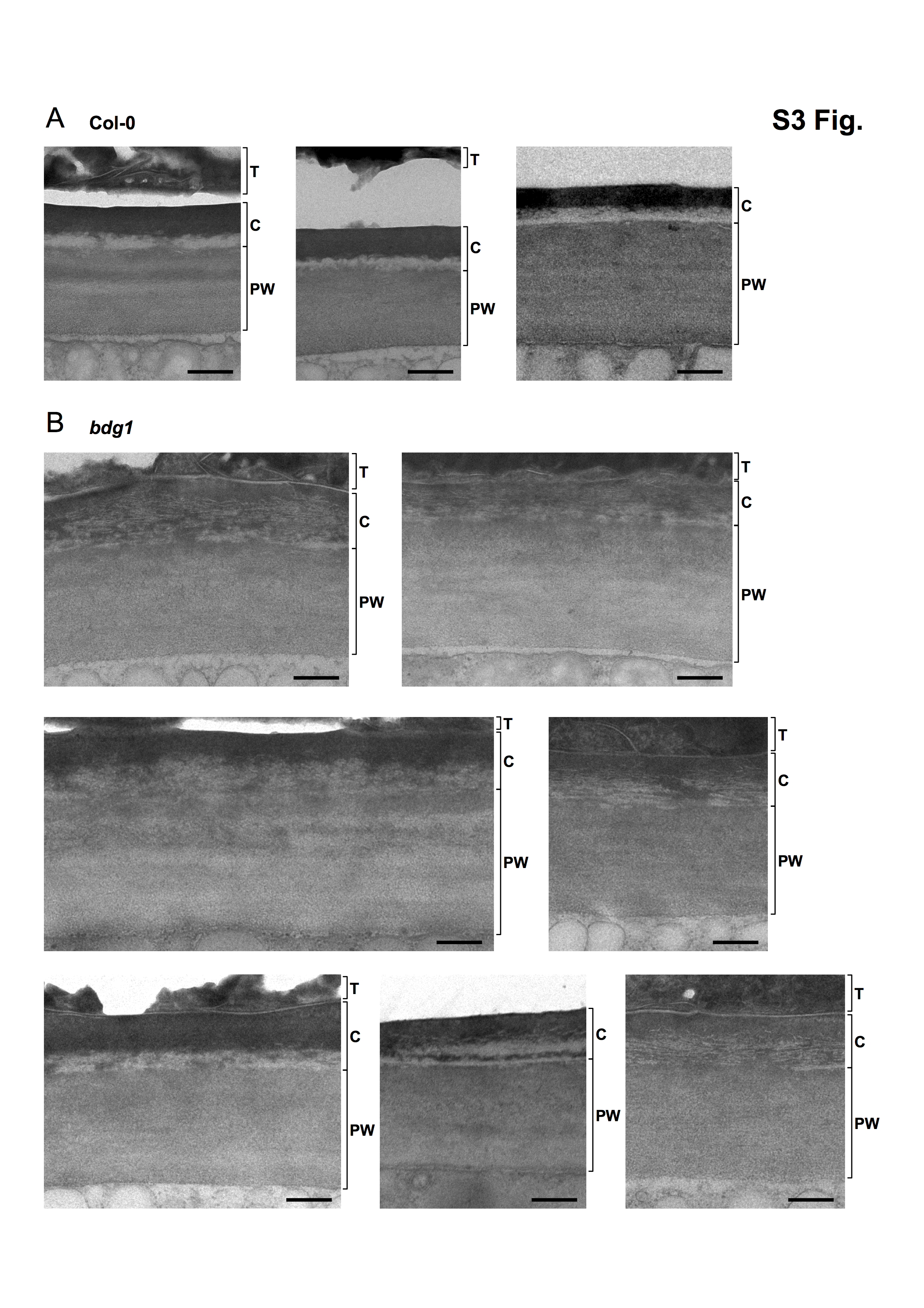

Supplement: S3 Fig — Close-ups of the outer side of endosperm cells in Col-0 seeds (A) and bdg1 seeds (B). Each electron micrograph represents a different cell, and a variety of cuticular defects are visible in bdg1 cells (B). Note that during the preparation of the specimens for electron microscopy, the testa sometimes gets detached from the endosperm and is sometimes not visible in proximity of the endosperm cell wall (A, right image; B, bottom middle image). Abbreviations are: T: testa; C: cuticle; PW: primary wall. Bars: 300nm. (TIFF) [file pgen.1005708.s003.tiff]

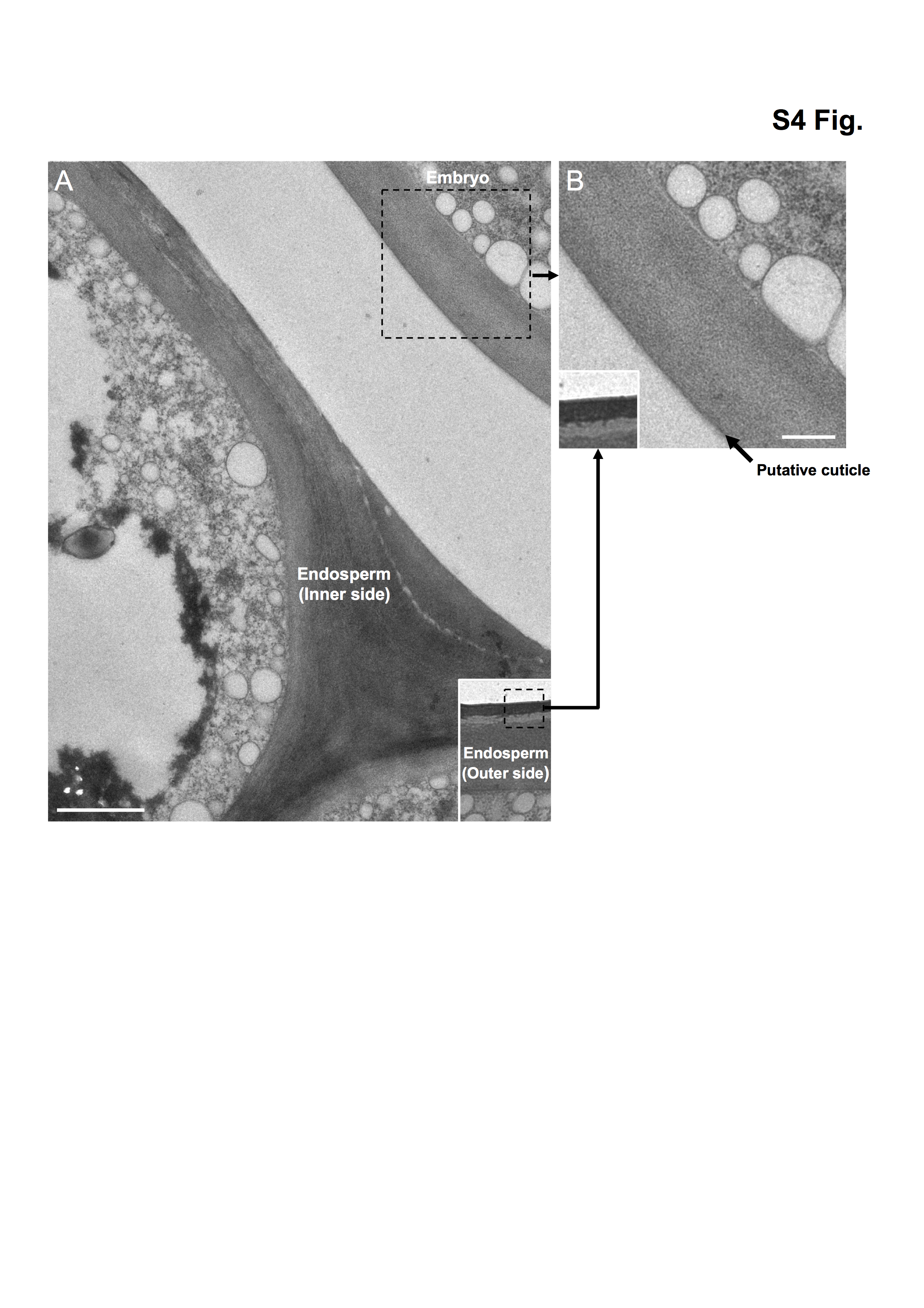

Supplement: S4 Fig — (A) Electron micrograph of a Col-0 endosperm cell (bottom left) and a neighboring embryonic cell (top right) having a potential cuticle. The inset in the bottom right corner shows, with the same magnification, the primary cell wall and the cuticle observed on the outer side of an endosperm cell in the same seed and on the same electron microscopy grid. Bar: 1000nm. (B) Close-up of the boxed area in (A). The inset in the bottom left corner shows a close-up of the boxed area in the inset in (A). Bar: 300nm. (TIFF) [file pgen.1005708.s004.tiff]

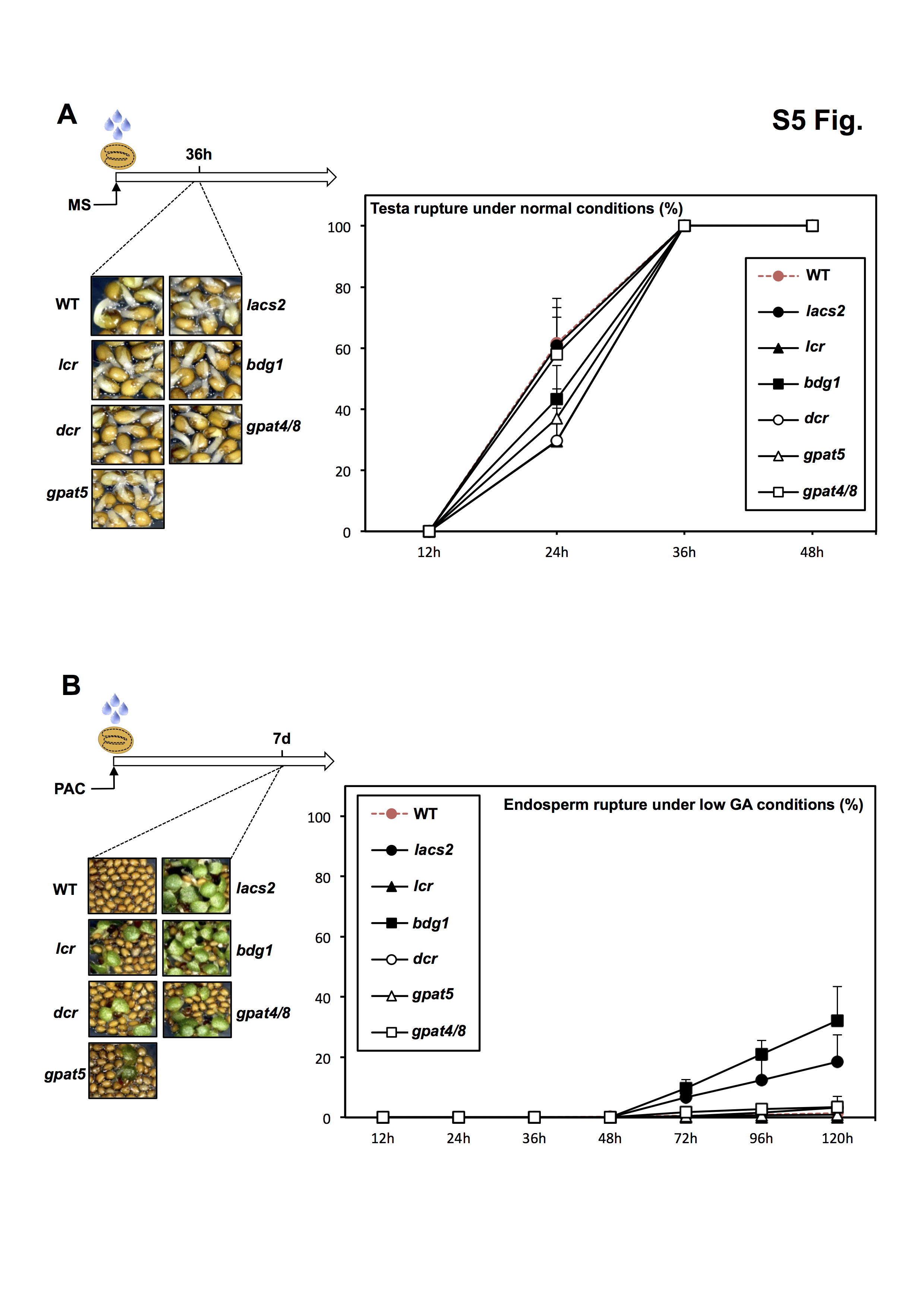

Supplement: S5 Fig — (A) Images showing WT (Col), lacs2, lcr, bdg1, dcr, gpat4/8, gpat5 seeds 36h after imbibition under normal germination conditions. Chart indicates percentage of testa rupture over time under normal germination conditions (two independent batches (n = 150–200)). (B) Images showing WT (Col), lacs2, lcr, bdg1, dcr, gpat4/8, gpat5 seeds 7days after imbibition under low GA conditions. Chart indicates percentage of endosperm rupture over time under low GA conditions (two independent batches (n = 150–200)). (TIFF) [file pgen.1005708.s005.tiff]

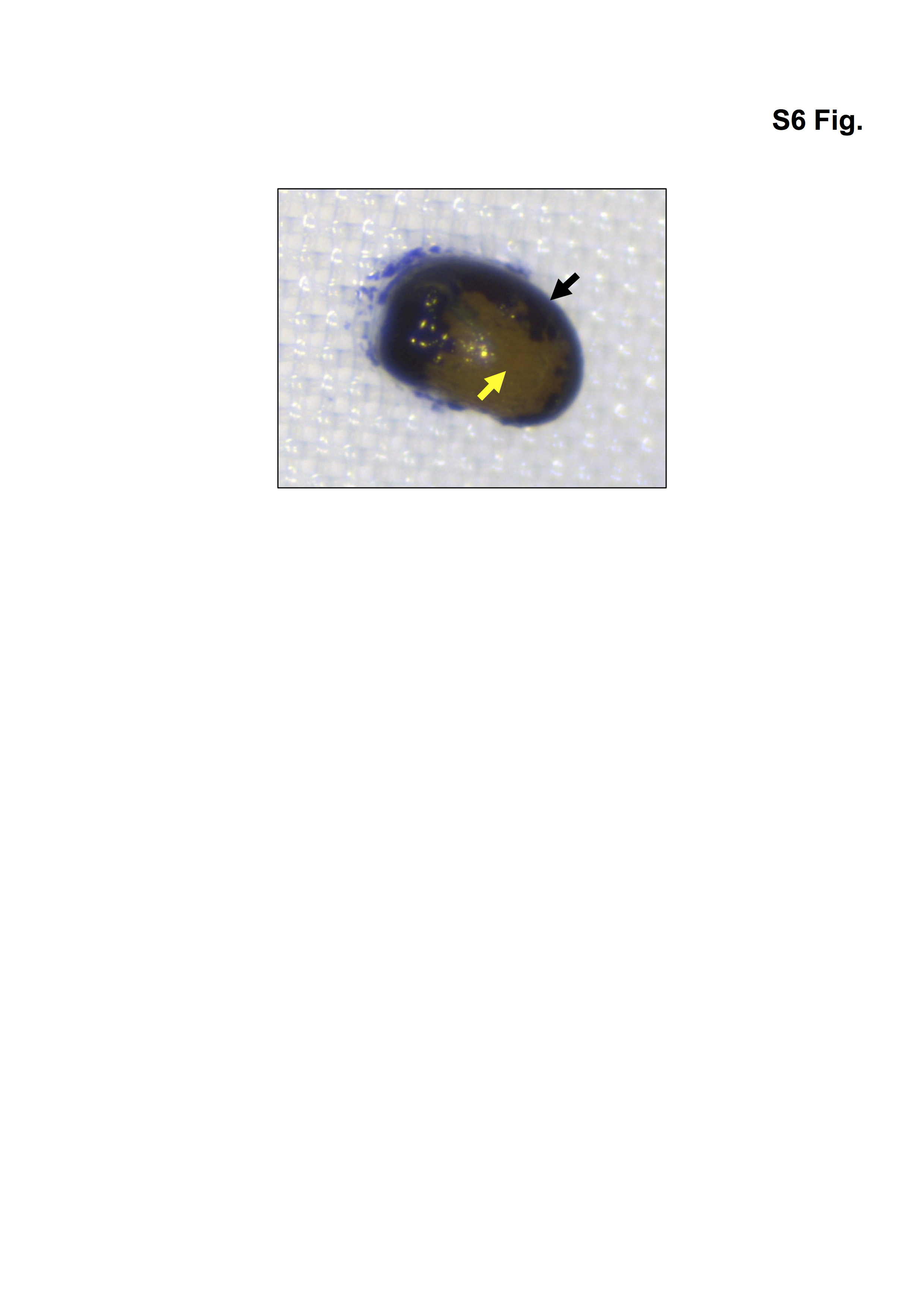

Supplement: S6 Fig — Image showing dissected WT seed coat (testa + endosperm) from PAC-treated seeds after 6 hours of toluidine blue incubation as in Fig 6. Yellow arrow indicates inner side of the seed coat (that includes the endosperm). Black arrow indicates the outer side of the seed coat. (TIFF) [file pgen.1005708.s006.tiff]

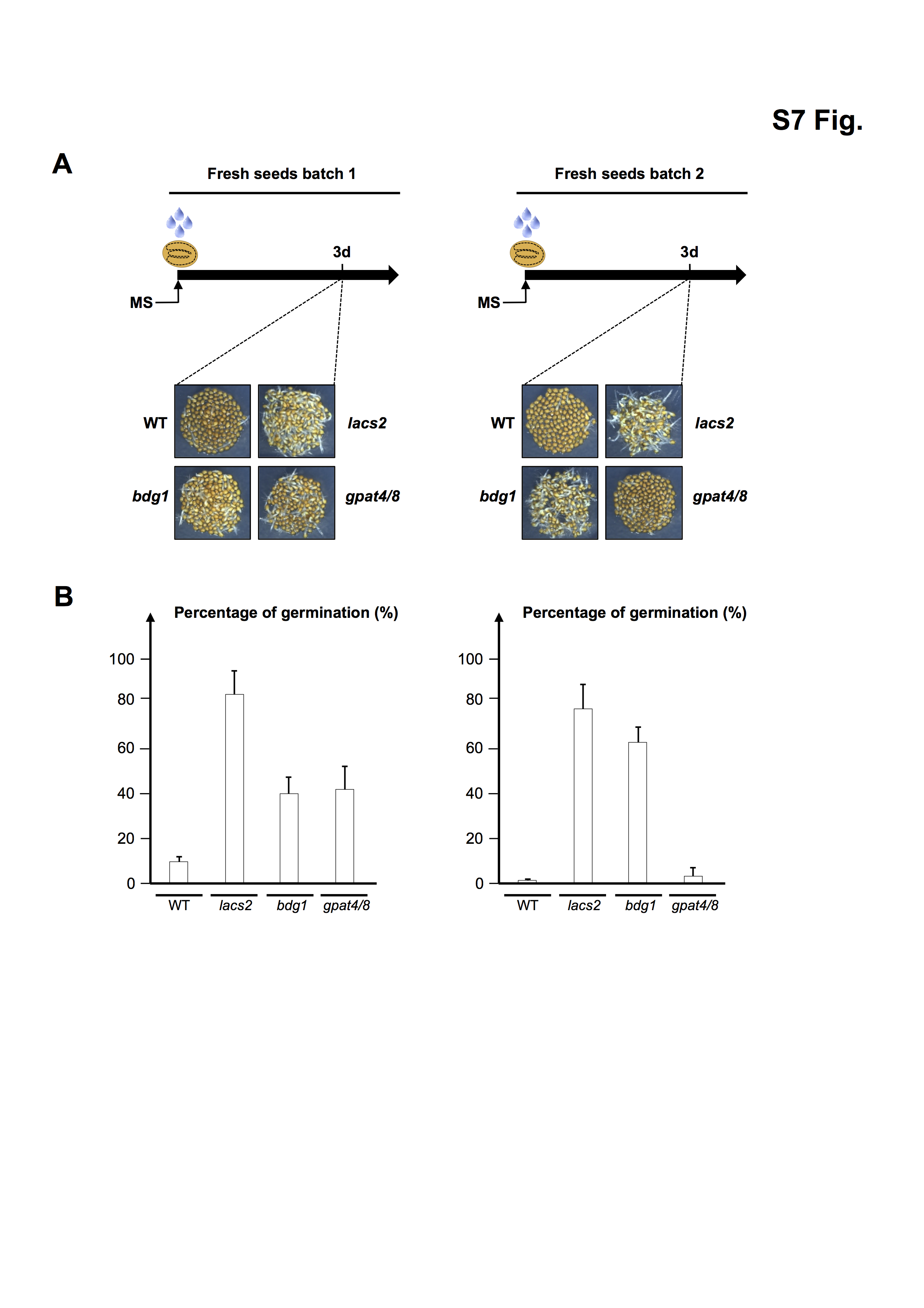

Supplement: S7 Fig — (A) WT (Col), lacs2, bdg1, gpat4/8 seeds seed germination in darkness was assessed 3 days after imbibition. (B) Histograms represent the percentage of germination of WT (Col), lacs2, bdg1 and gpat4/8 seeds 3 days after imbibition in darkness (For each independent seed batches seeds were plated in duplicate (n = 100–150)). (TIFF) [file pgen.1005708.s007.tiff]

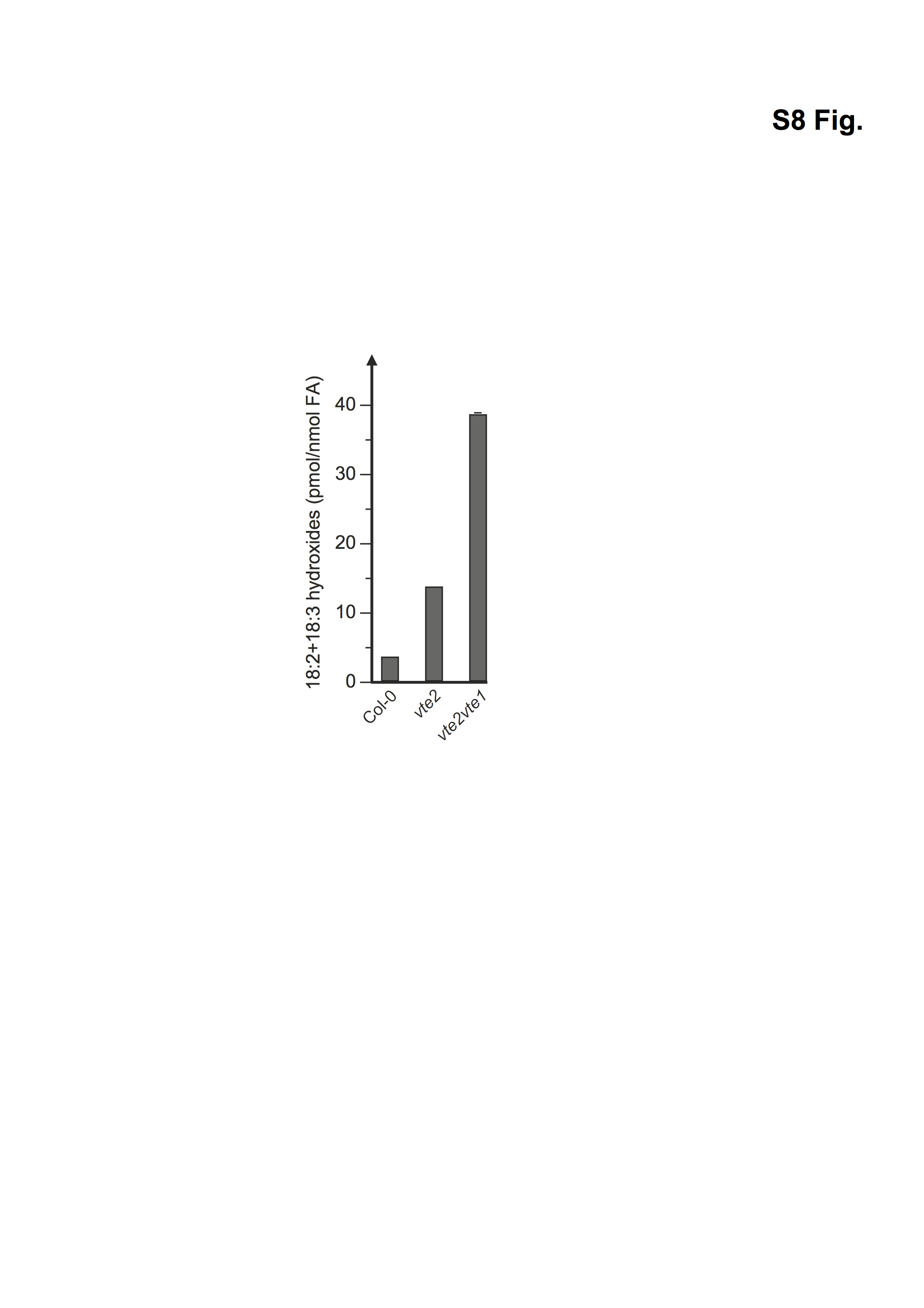

Supplement: S8 Fig — (TIFF) [file pgen.1005708.s008.tiff]

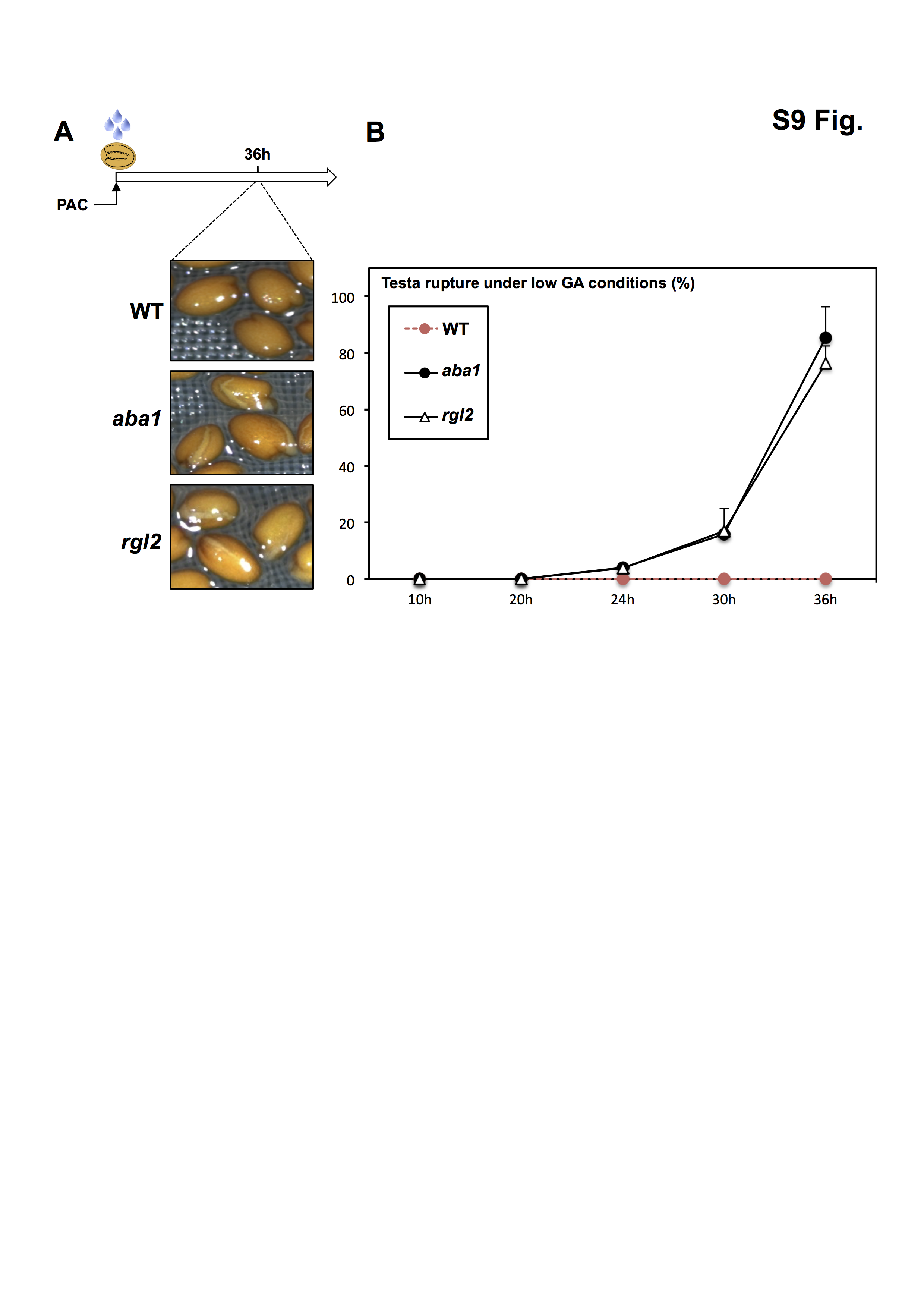

Supplement: S9 Fig — (A) Images showing WT (Col), aba1 and rgl2 seeds 36h after imbibition under low GA conditions. (B) Chart represents percentages of testa rupture over time (in hours) under low GA conditions (3 replicates (n = 50–100)). (TIFF) [file pgen.1005708.s009.tiff]

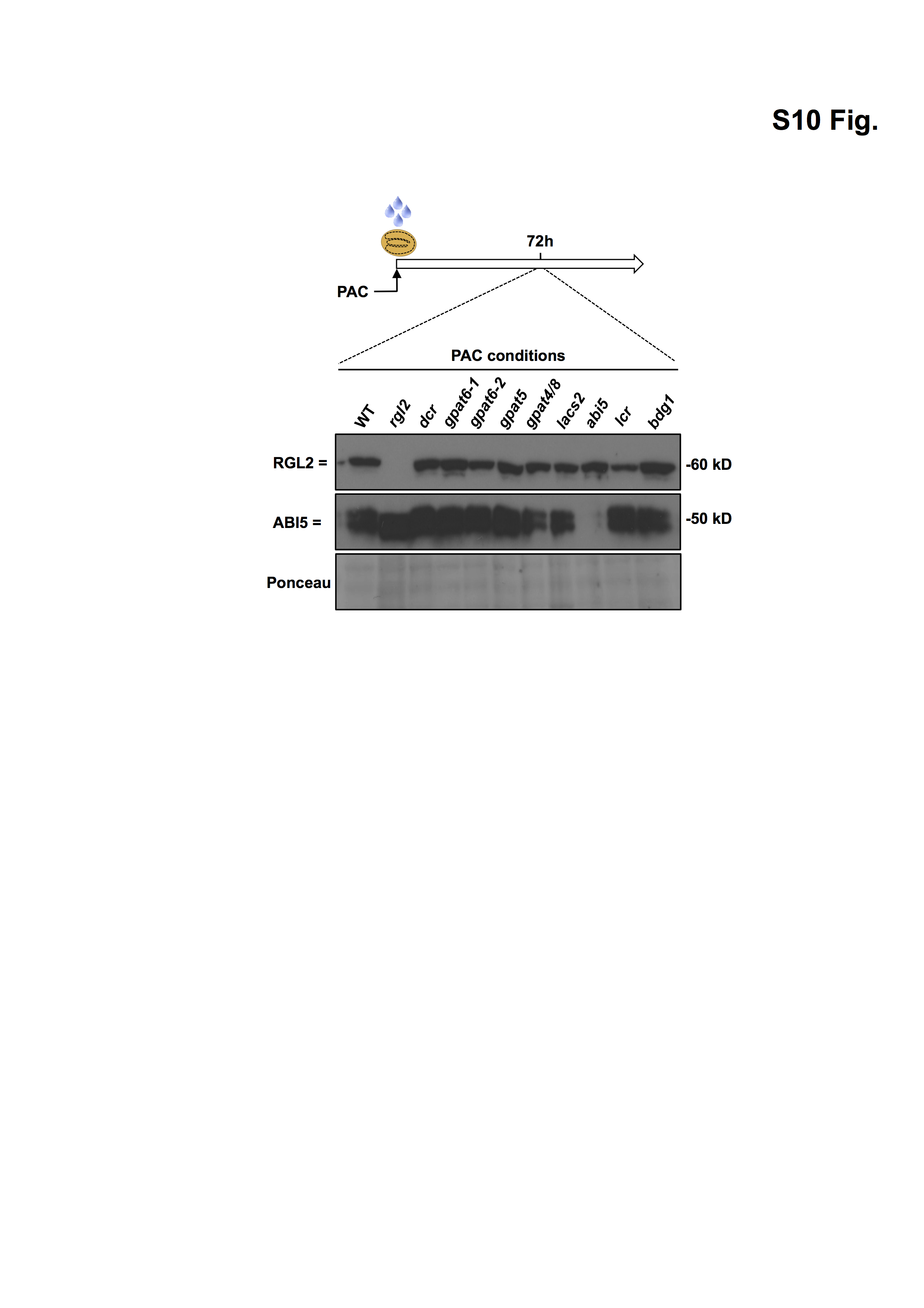

Supplement: S10 Fig — Protein gel blot analysis of RGL2 and ABI5 proteins. Ten micrograms of total proteins extracted from seeds harvested at 72 h after imbibition in presence of PAC was used per lane. Protein extracts were stained with Ponceau S as a loading control prior to detection with antibodies against RGL2 and ABI5. (TIFF) [file pgen.1005708.s010.tiff]

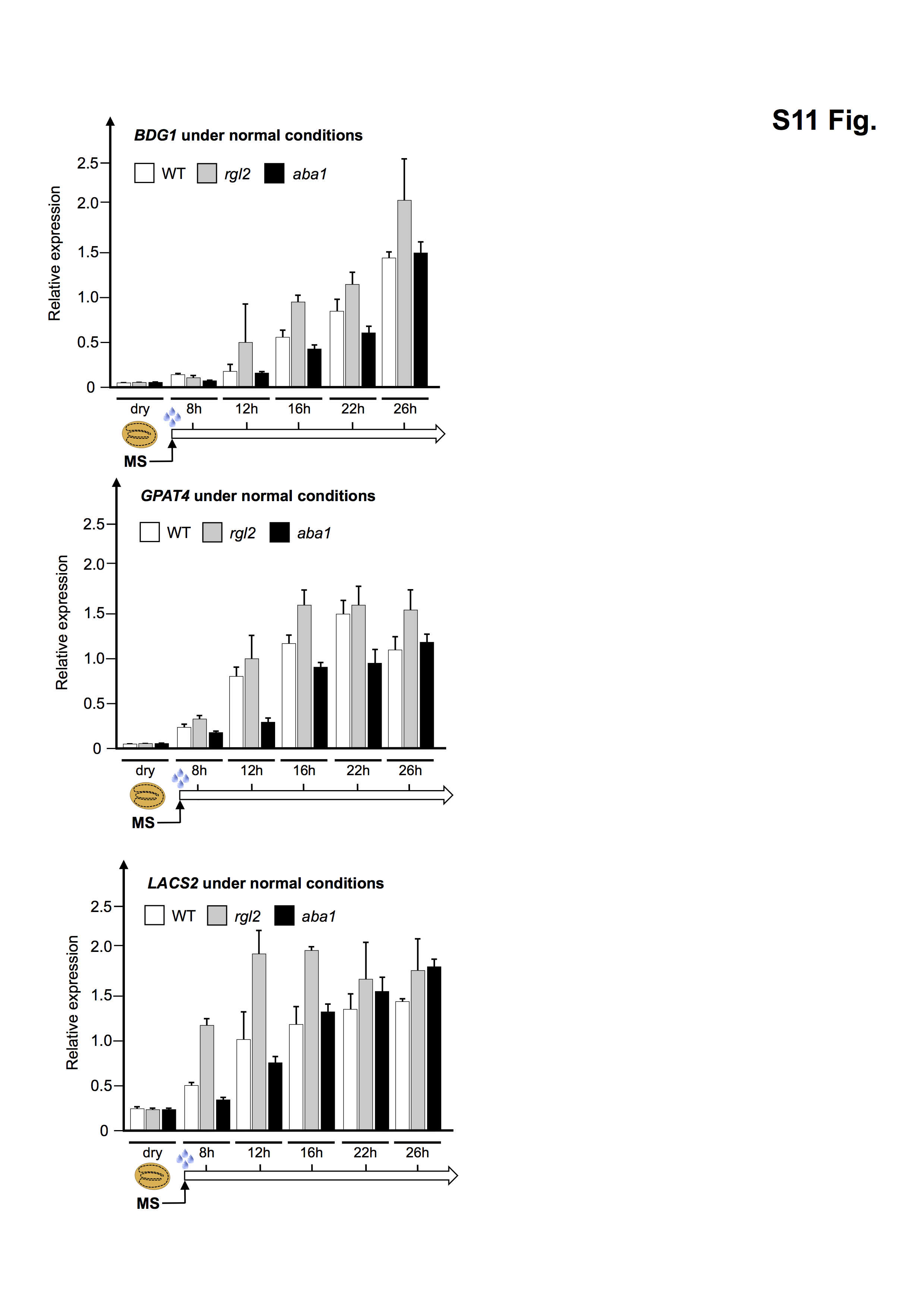

Supplement: S11 Fig — Histograms show the relative BDG1, GPAT4 and LACS2 mRNA accumulation in dry seeds and upon seed imbibition under normal germination conditions in WT (Col) as well as in rgl2 and aba1 mutants. (TIFF) [file pgen.1005708.s011.tiff]

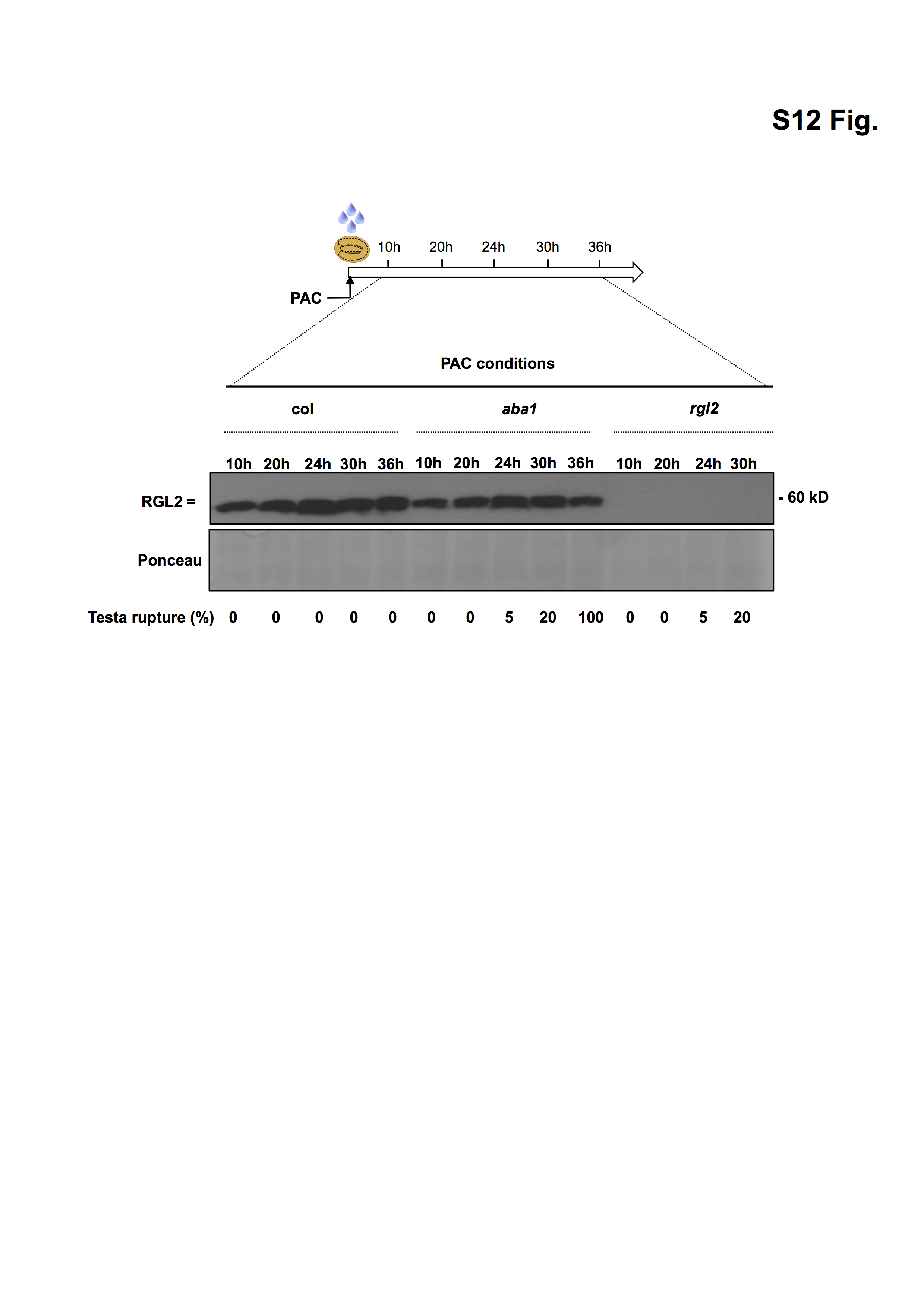

Supplement: S12 Fig — Protein gel blot analysis of RGL2 protein. Ten micrograms of total protein was used per lane. Protein extracts were stained with Ponceau S as a loading control prior to detection with antibodies against RGL2. Percentage of testa rupture is indicated under each time point. (TIFF) [file pgen.1005708.s012.tiff]

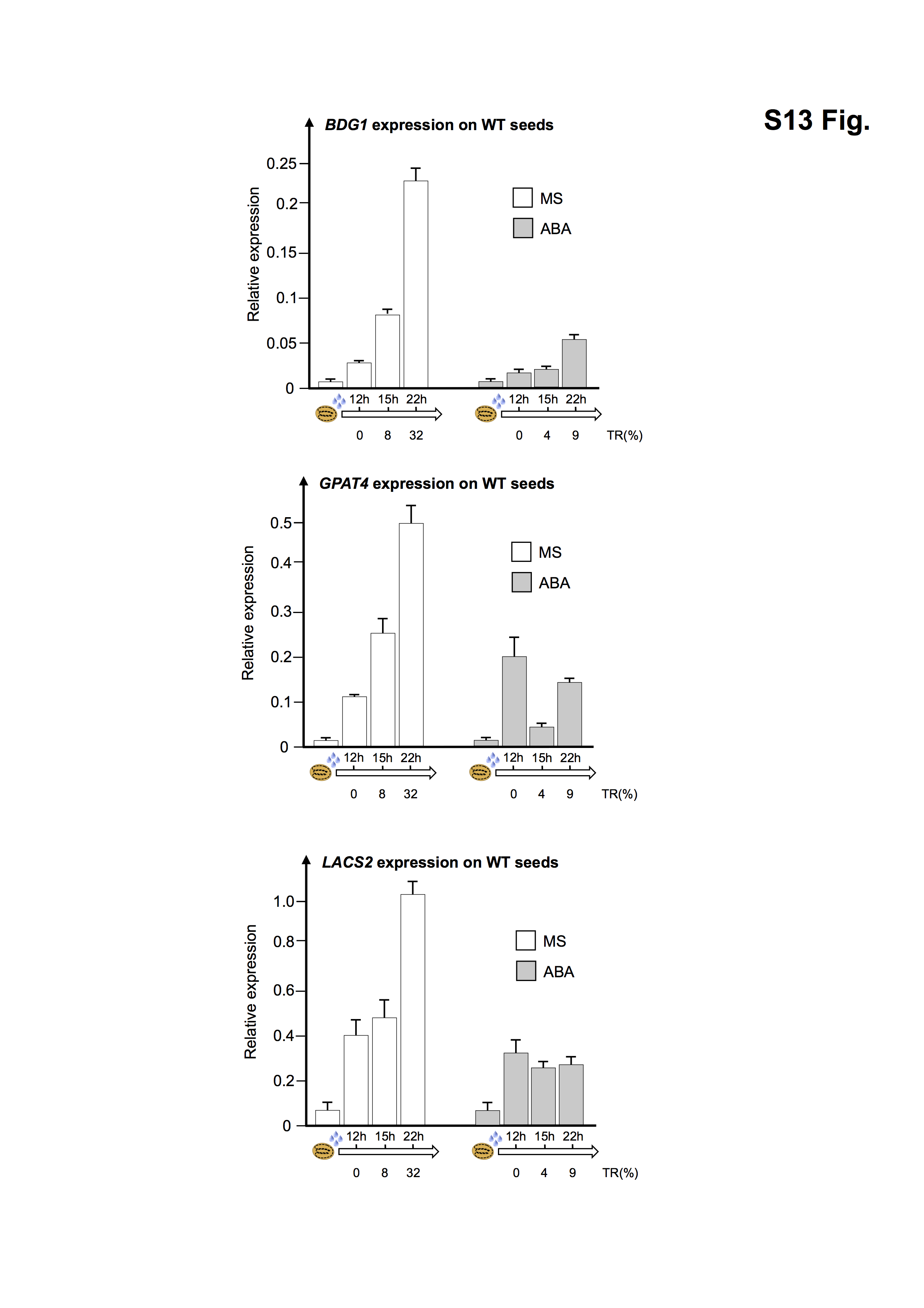

Supplement: S13 Fig — Histograms show the relative BDG1, GPAT4 and LACS2 mRNA accumulation in WT (Col) dry seeds and upon seed imbibition in absence (MS) and presence (ABA) of exogenous ABA. Expression of these genes coincides with the testa rupture events. (TIFF) [file pgen.1005708.s013.tiff]

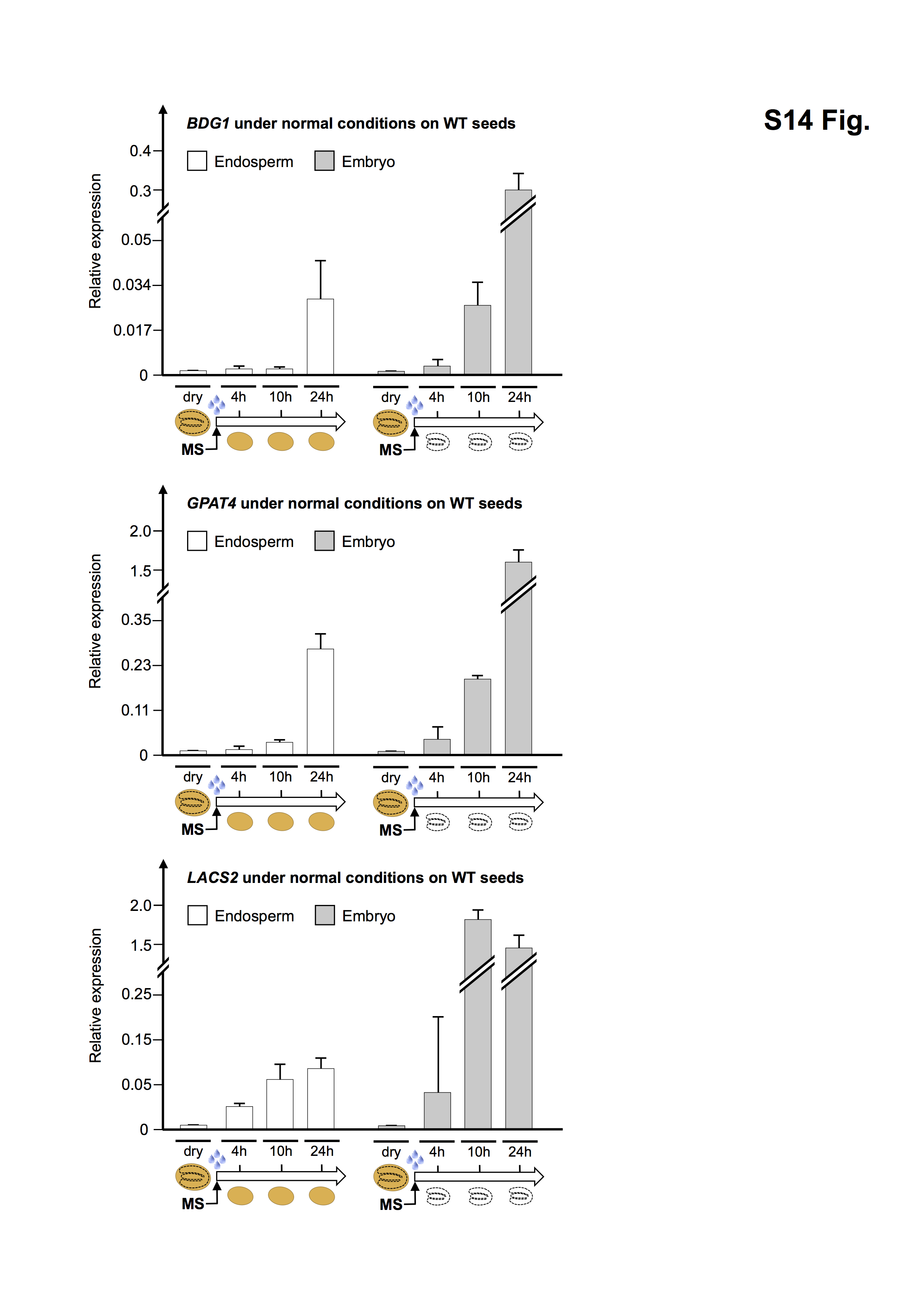

Supplement: S14 Fig — Histograms show the relative BDG1, GPAT4 and LACS2 mRNA accumulation in both endosperm and embryo of WT (Col) seeds imbibed under normal germination conditions. The expression in dry seed (dry) is also shown. (TIFF) [file pgen.1005708.s014.tiff]

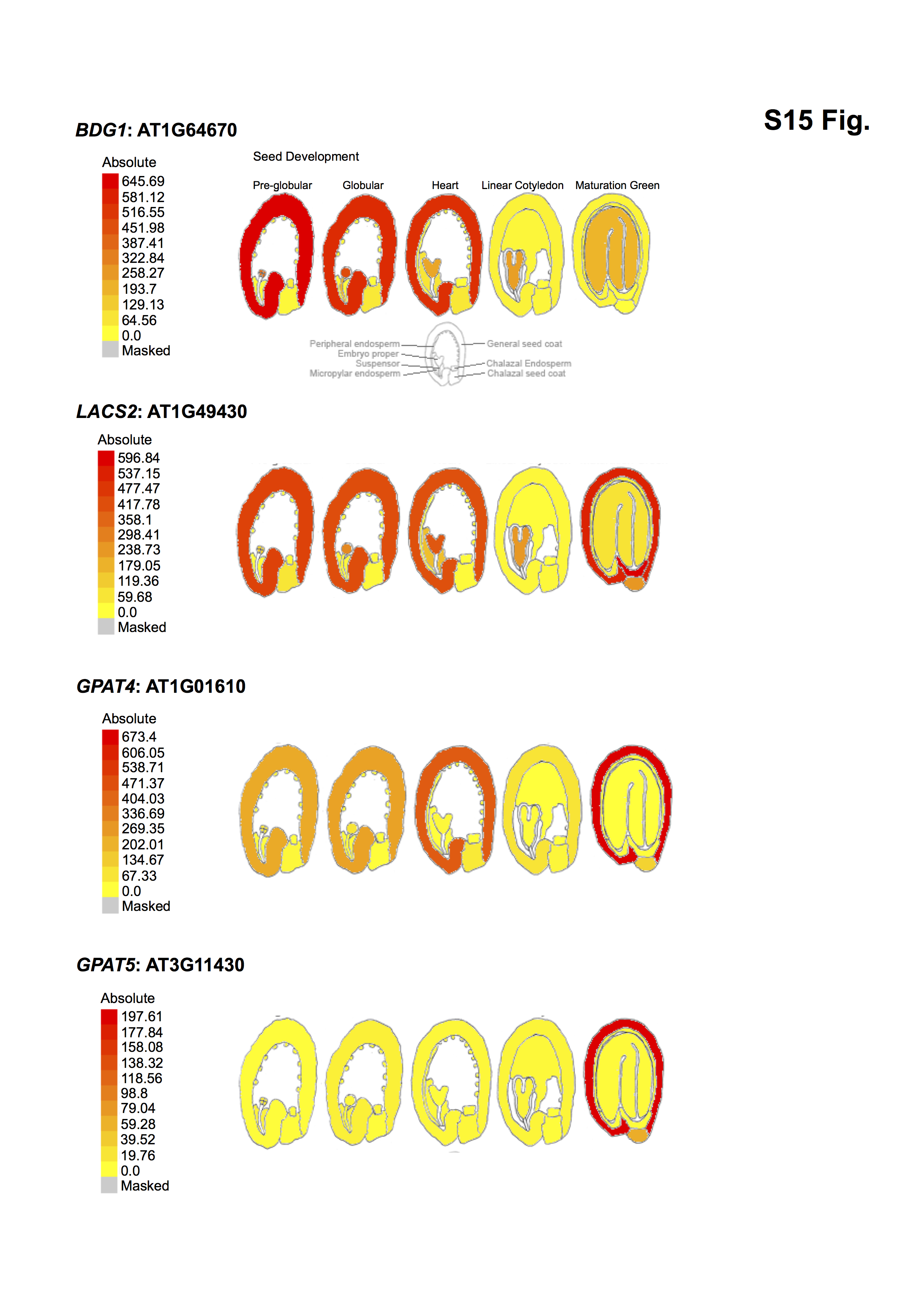

Supplement: S15 Fig — Data shown were retrieved from the Arabidopsis eFP browser website (http://bar.utoronto.ca/efp/cgi-bin/efpWeb.cgi). Original data published in Le et al 2010. Winter et al. PLoS One. 2007 Aug 8;2(8):e718; Le et al PNAS 2010 May 4;107(18):8063–70 (TIFF) [file pgen.1005708.s015.tiff]
