# Supplementary material for: An Endosperm-Associated Cuticle Is Required for Arabidopsis Seed Viability, Dormancy and Early Control of Germination
Source: PLoS Genet. 2015 Dec 17;11(12):e1005708. doi: 10.1371/journal.pgen.1005708 (PMC4683086; doi:10.1371/journal.pgen.1005708)
Supplement: S1 File — (PDF) [file pgen.1005708.s018.pdf]

University of Geneva – iGE3 Genomics Platform - CMU
L. Lopez Molina, Microarray: Time-course May 2010
second analysis January 2015

Microarray Affymetrix Arabidopsis ATH1 Genome Array (ATH1-121501)

Experimental design:

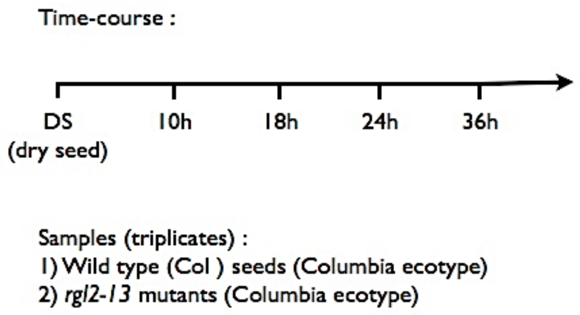

The samples were RMA normalized. The intensities before normalization are shown left and after normalization right. The normalized intensities are provided in text format files.

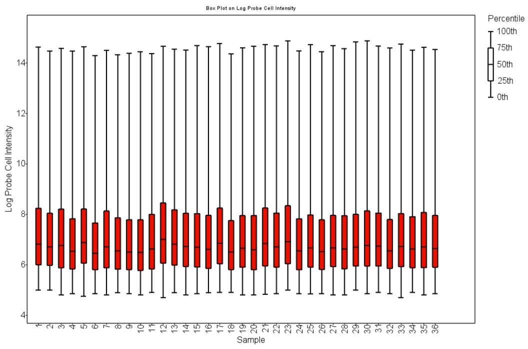

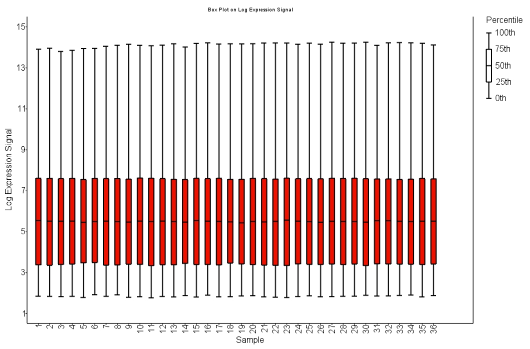

Differentially expressed genes :

The following tables summarizes the number of differentially expressed genes per comparison, with a FDR (false discovery rate) correction of 5% and a fold change >2 or <-2.

	Number of differentially expressed genes vith a FDR 5% and fold change < -2 or > 2		
**comparison:**	**# vary**	**# FC > 2 (upregulated)**	**# FC < -2 (downregulated)**
**rgl2 versus Col at 10h**	45	14	31
**rgl2 versus Col at 18h**	91	52	39
**rgl2 versus Col at 24h**	213	147	66
**rgl2 versus Col at 36h**	839	457	382

The files containing the annotated results are provided in excel format.

PCA :

The PCA (principal component analysis) of the samples is shown. The same PCA is colored according to the time factor (first figure, above) and according to the genotype (second figure, below). The samples separate according to the genotype (sample) and the time.

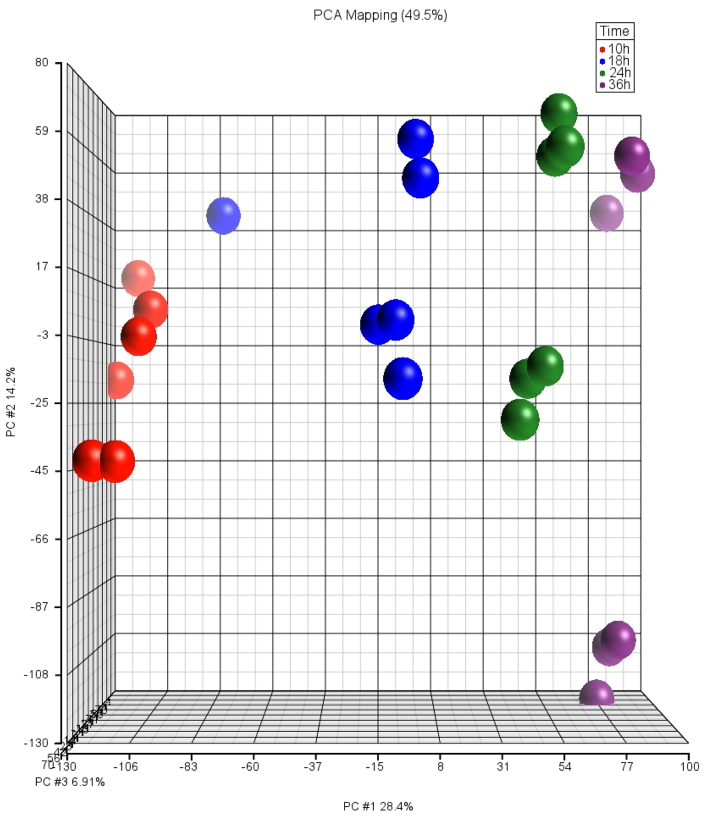
PCA Mapping (49.5%)
Time
10h
18h
24h
36h
PC #2 14.2%
80
59
38
17
-3
-25
-45
-66
-87
-108
-130
-130
-106
-83
-60
-37
-15
8
31
54
77
100
PC #3 6.91%
PC #1 28.4%

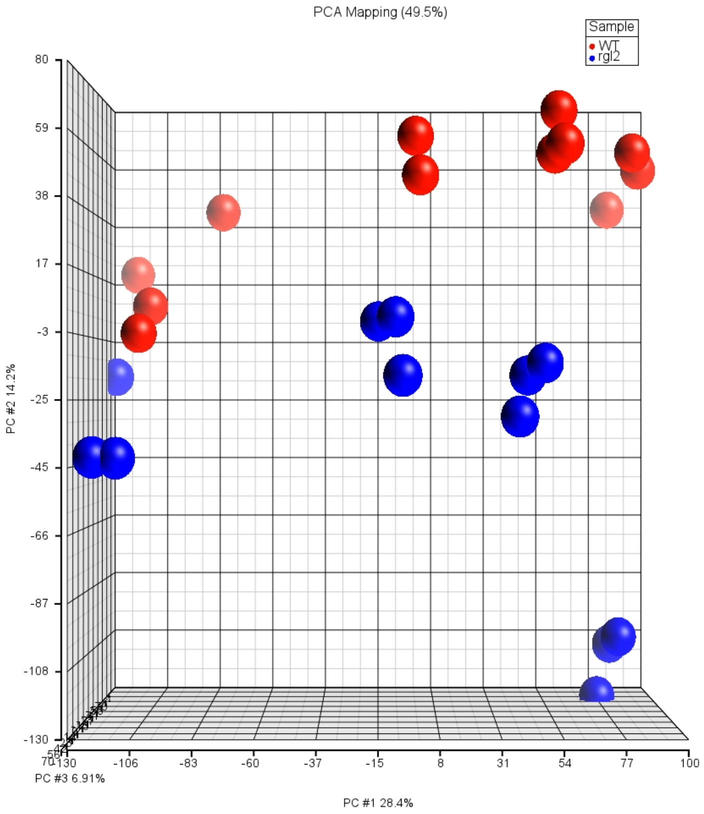
PCA Mapping (49.5%)
Sample
WT
rgl2
PC #2 14.2%
80
59
38
17
-3
-25
-45
-66
-87
-108
-130
-130
-106
-83
-60
-37
-15
8
31
54
77
100
PC #3 6.91%
PC #1 28.4%

The following plot shows the sources of variation. It is consistent to the PCA. All the factors in the ANOVA model are listed on the X- axis including random error. The Y- axis represents the average mean square of all the probesets. Mean square is ANOVA's measure of variance. Each signal bar has to be compared to the error bar; if a bar is higher than the error bar it means that the factor contributed significant variation to the data across all the probesets.

In this data set, the Time factor followed by the Sample factor have the biggest effect.

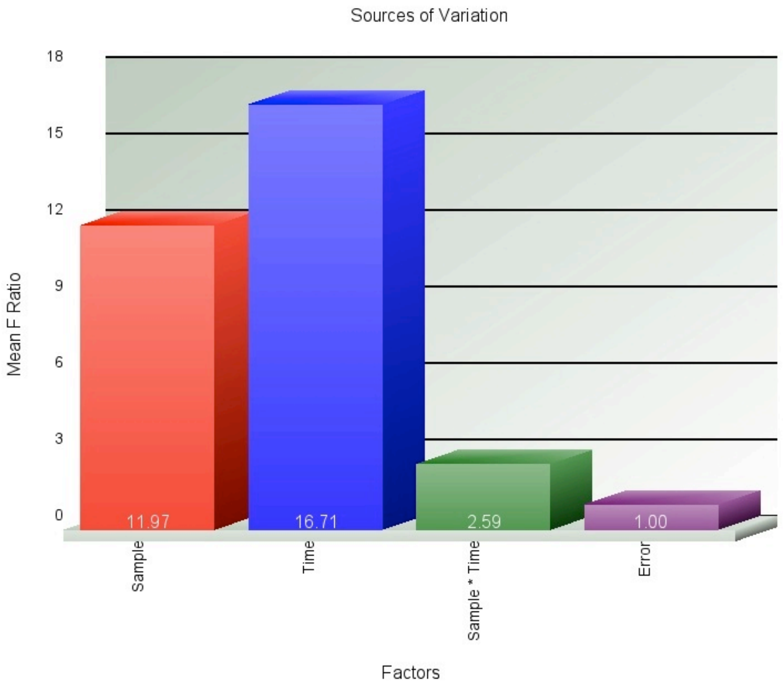

Venn diagram

The following figure is a Venn diagram representation of all the significantly differentially expressed genes. The relevant individual gene lists are provided upon request.

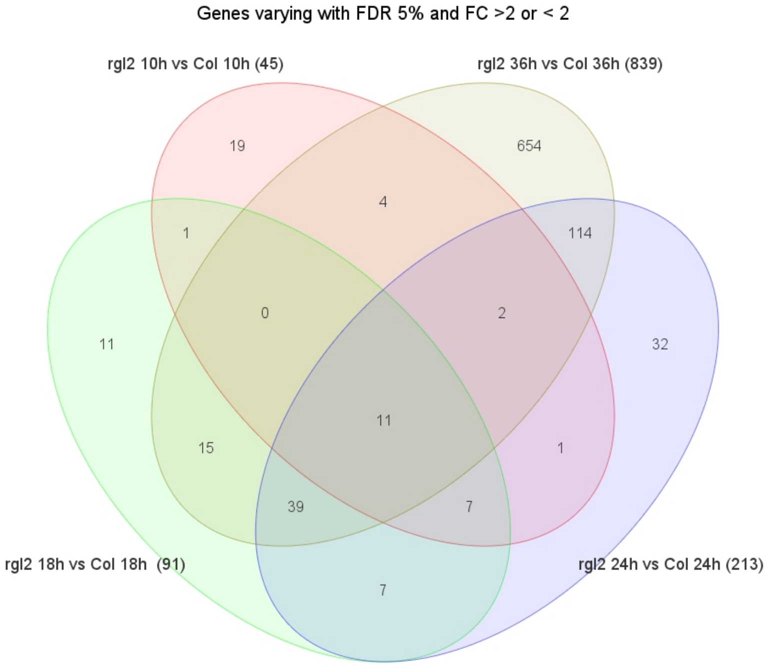

<u>**References :**</u>

RMA normalization: Bolstad BM, Irizarry RA, Astrand M, Speed TP. A comparison of normalization methods for high density oligonucleotide array data based on bias and variance. Bioinformatics 2003;19:185–193.

FRD correction : Hochberg Y, Benjamini Y. More powerful procedures for multiple significance testing. Stat Med 1990;9:811–818

Partek analysis software : Partek® Genomics Suite® software, version 6.6. Partek Inc., St. Louis, MO, USA.
